# DNA methylation and brain structure and function across the life course: A systematic review

**DOI:** 10.1016/j.neubiorev.2020.03.007

**Published:** 2020-06

**Authors:** Emily N.W. Wheater, David Q. Stoye, Simon R. Cox, Joanna M. Wardlaw, Amanda J. Drake, Mark E. Bastin, James P. Boardman

**Affiliations:** a Medical Research Council Centre for Reproductive Health, University of Edinburgh, United Kingdom; b Department of Psychology, University of Edinburgh, United Kingdom; c Centre for Clinical Brain Sciences, University of Edinburgh, United Kingdom; d University/British Heart Foundation Centre for Cardiovascular Science, University of Edinburgh, United Kingdom

**Keywords:** DNA methylation, Magnetic resonance imaging, Brain, Epigenetics

## Abstract

MRI has enhanced our capacity to understand variations in brain structure and function conferred by the genome. We identified 60 studies that report associations between DNA methylation (DNAm) and human brain structure/function. Forty-three studies measured candidate loci DNAm; seventeen measured epigenome-wide DNAm. MRI features included region-of-interest and whole-brain structural, diffusion and functional imaging features. The studies report DNAm-MRI associations for: neurodevelopment and neurodevelopmental disorders; major depression and suicidality; alcohol use disorder; schizophrenia and psychosis; ageing, stroke, ataxia and neurodegeneration; post-traumatic stress disorder; and socio-emotional processing. Consistency between MRI features and differential DNAm is modest. Sources of bias: variable inclusion of comparator groups; different surrogate tissues used; variation in DNAm measurement methods; lack of control for genotype and cell-type composition; and variations in image processing. Knowledge of MRI features associated with differential DNAm may improve understanding of the role of DNAm in brain health and disease, but caution is required because conventions for linking DNAm and MRI data are not established, and clinical and methodological heterogeneity in existing literature is substantial.

## Highlights

• 60 studies of association between DNAm and human brain MRI were identified.
• Differential DNAm is associated with brain structure and function throughout life.
• There is modest consistency between DNAm and image endophenotypes.
• Approaches for reducing heterogeneity in DNAm-MRI analyses are proposed.

## Nomenclature

**Abbreviations**
ACR: anterior corona radiata
AD: alcohol dependence
AxD: axial diffusivity
ADC: apparent diffusion coefficient
aMCI: amnesic mild cognitive impairment
BCC: body of the corpus calllosum
CC: corpus callosum
CLASP: Constrained Laplacian-based Automated Segmentation with Proximities
CSF: cerebral spinal fluid
CST: corticospinal tract
DG: dentate gyrus
DMR: differentially methylated region
FA: fractional anisotropy
FXTAS: Fragile X Associated Tremor Ataxia Syndrome
GCC: genu of corpus callosum
GM: grey matter
HC: healthy controls
HS: hippocampal sclerosis
IFG: inferior frontal gyrus
IT: inferior temporal
MD: mean diffusivity
MDD: major depressive disorder
MFG: middle frontal gyrus
mOFC: medial orbitofrontal cortex
MRF: Markov Random Field
MTLE: mesial temporal lobe epilepsy
NAcc: nucleus accumbens
NR: not reported
PCR: posterior corona radiata
PHC: parahippocampal cingulum
PNT: probabilistic neighbourhood tractography
PTR: posterior thalamic radiation
RD: radial diffusivity
RLP: right lateral parietal area
RS: resting state
SFG: superior frontal gyrus
STG: superior temporal gyrus
TBSS: tract-based spatial statistics
vlPFC: ventrolateral prefrontal cortex
vlThalamus: ventrolateral thalamus
WB: whole brain
WMH: white matter hyperintensities

**Gene names**
SKA2: Spindle and Kinetochore Associated Complex Subunit 2
*SLC6A4*: Solute Carrier Family 6 Member 4
*BDNF*: Brain Derived Neurotrophic Factor
*FKBP5*: FK506 Binding Protein 51
*OXTR*: Oxytocin Receptor
*NR3C1*: Nuclear Receptor Subfamily 3 Group member 1
*FMR1*: Fragile X Mental Retardation 1
*COMT*: Catecholamine Transferase
*DAT/SLC6A3*: Dopamine Transporter/Solute Carrier Family 6 Member 3
*OXT*: Oxytocin/Neurophysin 1 Prepropeptide
*C9orf72*: Chromosome 9 Open Reading Frame 72
*CACNA1C*: Calcium Voltage-Gated Channel Subunit Alpha1 C
*DRD2*: Dopamine Receptor 2
*KLF13*: Kruppel Like Factor 13
*NCAPH2*: Non-SMC Condensin II Complex Subunit H2
*LMF2*: Lipase Maturation Factor 2
*PPM1G*: Protein Phosphatase Magnesium Dependent 1 Gamma
*HTR3A*: 5-Hydroxytryptamine Receptor 3A
*SOD*: Superoxide Dismutase
*TESC*: Tescalcin

## 1 Introduction

A growing number of studies have investigated associations between epigenetic signatures and neuroimaging markers of human disease, behaviour and cognition. This area of research is motivated by the understanding that epigenetic processes contribute to brain development, ageing and disease, and they may mediate interaction between genomic predisposition and environmental pressures that modify brain structure and function.

The term epigenetics refers to a set of molecular mechanisms that modulate the function of the genome in different cell types without altering the genome itself. DNA methylation (DNAm) is one such mechanism whereby a methyl group is covalently added to cytosine residues in a Cytosine-phosphate-Guanine context (CpG). This is thought to alter the accessibility of a locus to transcriptional machinery or modifying proteins, thereby influencing gene expression. DNA methylation may also occur after an initial change in gene expression and function as a form of longer-term control (Jones, 2012).

The pathogenesis of several genetic diseases with neurological phenotypes involve DNAm dysregulation. Rett syndrome is a severe neurological disorder characterised by regression of acquired skills, stereotypic movements, microcephaly, seizures, and intellectual disability. It results from a loss of function mutation in the X-linked methyl-CpG binding protein 2 gene (*MECP2*), which is a chromatin associated protein required for normal neuronal function throughout life. The loss of function mutation in *MECP2* serves to reduce the binding affinity of the MECP2 protein to methylated DNA with effects on regulation of gene expression at transcriptional and post-transcriptional levels (Lyst and Bird, 2015). Angelman syndrome is characterised by cognitive impairment, movement or balance disorder, typical abnormal behaviours, and impaired speech and language. It arises from a mutation or deletion of the maternal ubiquitin protein ligase 3A gene (*UBE3A*) and the neuronal tissue specific paternal imprinting of the gene that silences the paternal allele (Rangasamy et al., 2013). Fragile X syndrome arises due to CGG triplet expansion within the *FMR1* gene promoter which becomes hypermethylated and results in a reduction of gene expression (Wijetunge et al., 2013). In addition to these genetic neurological disorders associated with DNAm dysregulation, variation in DNAm has been implicated in the pathogenesis of a number of complex neurodevelopmental, neurological and psychiatric diseases including autism, schizophrenia, and problems associated with trauma and stress (De Rubeis et al., 2014; Grayson and Guidotti, 2013; Klengel et al., 2014; Oberlander et al., 2008; Palumbo et al., 2018).

DNAm may be modifiable by environmental factors including physiological and emotional stress, child abuse, nutritional deprivation, and other lifestyle factors that operate from fetal life to old age (Cao-Lei et al., 2015; Del Blanco and Barco, 2018; Gouin et al., 2017; Heijmans et al., 2008; Joehanes et al., 2016; McGowan et al., 2009; Provenzi et al., 2018; Vidal et al., 2014). As such, it has been proposed that environmentally-induced changes in DNAm may enable short-term survival adaptation, but may also induce adaptations that contribute to impaired development of neural networks and increased risk of pathology (Gluckman et al., 2010; Hoffmann et al., 2017).

Structural, diffusion and functional brain MRI enable the parsing of complex behavioural traits and diseases onto quantitative indicators of brain structure and function. Such imaging endophenotypes have been used to demonstrate the impact of common genetic variants on brain health and disease (Elliott et al., 2018), but the role of DNAm in contributing to brain structure and / or function, as opposed to being a consequence of changes in structure or function, is less certain. To obtain a comprehensive overview of the extent to which differential DNAm associates human behaviour and disease we performed a systematic review of studies that have analysed DNAm with quantitative brain MRI data. By doing so we aimed to provide a comprehensive summary of what is known about DNAm-MRI associative relationships across the life course, and to identify image features associated with differential DNAm. Finally, we captured sources of heterogeneity in the extant literature to better inform the development of methods and conventions for analysing DNAm with MRI data.

## 2 Materials and methods

We performed a systematic literature search based on the PRISMA framework, according to a pre-registered protocol on PROSPERO (CRD42018090928).

### 2.1 Search strategy

Scopus, Web of Science, MEDLINE and EMBASE (via OVID) were searched in March 2018 to identify studies that integrated DNA methylation and human in vivo MRI (for search strategy see supplementary material). The search was composed of three OR clusters that contained terms relating to the key domains, which were combined with AND: MRI neuroimaging AND Brain AND DNA methylation. Bibliographies were searched for additional studies. There were no language restrictions on the search.

### 2.2 Screening and study selection

Search results were imported to EndNote X8 prior to removal of duplicates and retrieval of full texts prior to import to Covidence software (https://www.covidence.org/home). Screening was carried out by ENWW and DQS independently and studies were included if they met the following criteria: report of primary results; recruited human participants of any age with in vivo neuroimaging and DNAm; investigated the relationship between brain imaging using MRI and epigenome-wide or loci specific DNAm. Exclusion criteria: no original data reported (reviews, abstracts, letters, and grey literature as defined by Grey Literature International Committee Guidance); no test of association between brain imaging findings and DNA methylation; analysis was carried out in animals or in post-mortem human tissue; DNAm-MRI analyses of central nervous system malignancy.

### 2.3 Data extraction

ENWW extracted data for all included studies and DQS duplicated extraction for a subset of 25 %. Extraction included: participant characteristics, study design, method of DNAm ascertainment, MRI modality and image feature(s) of interest, and statistical method, consideration of cell sub-type composition includes cell sorting techniques or statistical methods to estimate and control for cell sub type composition and consideration of genotype or ancestry. The total number of participants per phenotypic category were estimated. Due to participant overlap in some studies, the largest study population reported was used to estimate the total number of participants.

### 2.4 Risk of bias assessment

In the absence of a validated quality assessment tool for studies linking DNAm with MRI data, we extracted data on study characteristics that might affect risk of bias: design, presence / absence of a comparator group; DNA source; candidate or epigenome-wide approach for evaluating differential DNAm; ascertainment of cell type composition; consideration of genotype; image processing methodology; and region-of-interest versus whole brain MRI analysis, and considered their prevalence in the epigenetic-neuroimaging literature.

### 2.5 Data synthesis

We provide a narrative synthesis structured around the type of association between DNAm and image feature, target population characteristics, tabulated, and categorised by disease.

## 3 Results

### 3.1 Overview of the literature

**Fig. 1:**
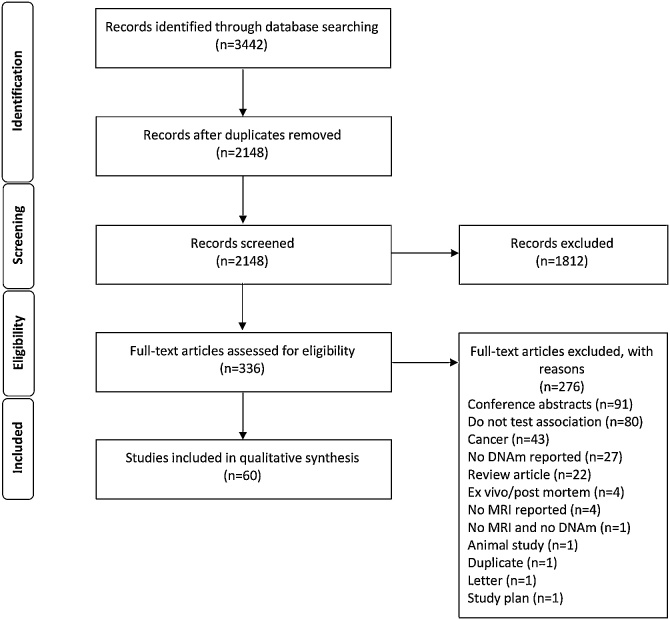
PRISMA flow diagram of studies.

Fig. 1 is the PRISMA flow chart of study identification and inclusion. Our search strategy yielded 3442 in total: 3438 resulting from the search and an additional 4 papers were identified post search and were included. After removal of duplicates and exclusions at screening, 336 articles were eligible for full text evaluation. Of the full text articles that were screened for eligibility for inclusion, 276 were removed due to lack of eligibility. In total, 60 studies were included in this review (estimated 6775 participants): 34 (57 %) included a control or comparator group. Table 1 summarises diseases and conditions in phenotypic categories, number of participants per category, MRI modality, and method used to analyse DNAm (epigenome wide or candidate loci). All studies report populations from resource rich settings: United States (25), United Kingdom (3), Germany (4), Switzerland (4), Germany and Switzerland (1), South Korea (7), Canada (3), Italy (2), Japan (1), China (2), Singapore (2), Republic of Ireland (2), Australia (2), Spain (1), Sweden (1).

**Table 1:** Overview of studies.

Disease or condition, number of studies	Number of participants	MRI modality (structural, functional, diffusion)	Epigenetic method: Epigenome-wide (EW) or candidate (candidate loci)
Neurodevelopment and neurodevelopmental disorders^†^ n = 8	715	5 sMRI 6 fMRI 2 dMRI	6 EW 2 candidate (*SLC6A4, FKBP5)*
Major depressive disorder and suicidality n = 11	922^§^	4 sMRI 4 fMRI 2 dMRI 1 sMRI/dMRI	1 EW 10 candidate (*BDNF, SLC6A4, FKPB5, CACNA1C, TESC)*
Alcohol use disorder n = 4	921	4 fMRI	0 EW 4 candidate *(PPM1G, DAT/SLC6A3, DRD2)*
Schizophrenia and psychosis n=7	456^§^	2 sMRI 4 fMRI 1 sMRI/fMRI	5 EW 3 candidate (*OXTR, BDNF, COMT)*
Ageing, stroke, ataxia and neurodegeneration n = 8	1456^§^	5 sMRI 2 dMRI 1 sMRI/dMRI	3 EW 5 candidate *(C9orf72, FMR1, SOD, NCAPH2/LMF2)*
Post-traumatic stress disorder n=7	450^§^	3 sMRI 3 fMRI 1 dMRI	1 EW 6 candidate *(SKA2, NR3C1, HTR3A, BDNF, FKBP5)*
Miscellaneous^‡^ n = 15	1855	3 sMRI 10 fMRI 2 sMRI/fMRI	1 EW 14 candidate *(COMT, OXTR, NR3C1, SLC6A4, OXT, FKBP5, KLF13, BDNF)*

^†^Includes studies of typical development, twin birth weight discordance, *BDNF* Val66 Met polymorphism, preterm birth, early life stress, attention deficit hyperactivity disorder, medial temporal lobe epilepsy.

^‡^Healthy adults, emotion processing, social anxiety disorder, Cushing’s syndrome.

^§^Estimate due to possible sample overlap within phenotypic category.

### 3.2 DNA methylation

All studies used surrogate tissues to probe methylation status: blood (n = 46); saliva (n = 12); one study reported using both blood and saliva, and one reported using blood/saliva/buccal cells (Ismaylova et al., 2017; Nikolova et al., 2014). The majority of studies did not report adjustments for cell type composition of samples used to estimate DNAm (n = 48).

Forty-three studies used a candidate gene analysis approach and 17 performed an epigenome-wide association study (EWAS). The majority of included studies that used a candidate gene approach measured methylation using bisulfite pyrosequencing (n = 36). Three studies measured methylation at a candidate gene using the EpiTYPER® system (Haas et al., 2016; Shelton et al., 2017, 2016). One study carried out a restriction enzyme digest and sequencing to measure DNAm at a candidate site (McMillan et al., 2015). Three studies measured DNAm using Illumina 450k arrays, and used the output to inform candidate gene DNAm-MRI analyses (Sadeh et al., 2016; Walton et al., 2014; Wiemerslage et al., 2017). Overall nineteen candidate loci were studied. *SLC6A4* (Na+/Cl- Dependent Serotonin Transporter) was the gene of interest in 11 studies; *BDNF* (member of nerve growth factor family), *FKBP5* (member of the immunophilin family) and *OXTR* (oxytocin receptor) were selected as candidate genes in four studies; *NR3C1* (glucocorticoid receptor) in three studies; *FMR1* (FMRP Translational Regulator 1), *COMT* (enzyme involved in catecholamine degradation) and *DAT/SLC6A3* (Na^+^/K^+^ dependent dopamine transporter) in two studies. The following genes were selected candidates in one study: *OXT* (encodes oxytocin and neurophysin 1 precursor protein)*, C9orf72* (thought to be a transcriptional regulator in the brain), *CACNA1C* (voltage gated calcium channel), *DRD2* (dopamine receptor), *KLF13* (transcription regulator), *NCAPH2/LMF2* (encoding both a non-SMC subunit of the condensin II complex and a lipase maturation factor protein from the same locus), *PPM1G* (member of the PP2C family of Ser/Thr protein phosphatases), *HTR3A* (member of the ligand-gated ion channel receptor superfamily), *SKA2* (Spindle And Kinetochore Associated Complex Subunit 2), *SOD* (superoxide dismutase) and *TESC* (tescalcin – regulates cell pH by controlling H+/Na + exchange across the plasma membrane).

All of the EWAS studies (n = 17) used Illumina Infinium arrays to measure DNAm: Illumina HumanMethylation27 (n = 6), Illumina 450k (n = 10), and Illumina EPIC (n = 1). These arrays measure methylation at 27 578, >450 000 and >850 000 CpG sites, respectively. Five studies investigated DNAm age, a method that uses a subset of CpG sites associated with age to investigate age and age acceleration (Chouliaras et al., 2018; Davis et al., 2017; Hodgson et al., 2017; Raina et al., 2017; Wolf et al., 2016).

Of studies which report DNAm at candidate genes, two reported that the sample used to measure DNAm was mononuclear blood cells (lymphocytes and monocytes) (Chouliaras et al., 2018; Ursini et al., 2011). None of the studies that measured DNAm by bisulfite pyrosequencing reported correction for cell type composition. 12 of the 20 studies which used Illumina arrays performed a correction for cell type composition. Only one study reported direct counting of cell types included in their samples (Freytag et al., 2017).

### 3.3 Magnetic resonance imaging

#### 3.3.1 MRI modalities

The majority of studies used 3 T MRI scanners (n = 42); five studies used 1.5 T scanners; five included participants scanned at 1.5 T and 3 T scanners; and eight studies did not report field strength. Many included studies carried out functional MRI (n = 29), of which three studies also carried out structural analysis. Structural MRI, to investigate volumes, cortical morphology, and conventional clinical MRI measures, was used in 26 studies. Diffusion MRI to measure properties of white matter microstructure was used in nine studies, and MR angiography was used in one.

#### 3.3.2 MRI features associated with DNAm across studies

Table 2 shows 10 MRI features that are associated with differential DNAm in two or more studies. The image features are: hippocampal volume (n = 11); hippocampal functional connectivity/activity (n = 4); amygdala functional connectivity/activity (n = 9); PFC functional connectivity/activity (n = 8); regional FA measures (n = 6); cortical thickness (n = 6); regional diffusion metrics (AxD/MD/RD) (n = 5); global FA (n = 2); and cortical volume (n = 2). For most of these, differential DNAm occurred at different loci. However, amygdala functional connectivity/activity was associated with differential DNAm in *SLC6A4* in six studies, and *OXTR* in two studies. PFC connectivity/activity was associated with DNAm in *BDNF* in two studies. Hippocampal volume was associated with *FKBP5* DNAm in two studies. Activation within the prefrontal cortex in working memory tasks were associated with *COMT* DNAm in two studies. The studies all employed a candidate gene approach to DNAm analysis and directions of association were mixed across the studies.

**Table 2:** Associations between image features and DNAm.

Image Feature (number of studies)	Differential DNAm: gene or feature
Global fractional anisotropy (n = 2)	DNAm age (Horvath) DNAm age (Hannum)
Regional fractional anisotropy (n = 6)	
• Corpus callosum	*SLC6A4* *FMR1* DNAm age (Hannum)
• Ventrolateral thalamus • Right anterior corona radiata • Right parahippocampal cingulum	*SMOC2* *BDNF* *TESC*
Regional radial, mean or axial diffusivity (n = 5) • Inferior and middle cerebellar peduncle • Corpus Callosum • Right anterior corona radiata • Right parahippocampal cingulum	*FMR1* DNAm age (Hannum) *SLC6A4* *BDNF* *TESC*
Amygdala functional connectivity (n = 9)	*SLC6A4* *SLC6A4* *SLC6A4* *SLC6A3/DAT* *SLC6A4* *OXTR* *OXTR* *SLC6A4* *SLC6A4*
Hippocampal functional connectivity (n = 4)	*SLC6A4* Cluster of genes identified through EWAS (Wang et al., 2017)* Cluster of genes identified through EWAS (Hu et al., 2018)* BDNF
Prefrontal cortex functional connectivity (n = 8)	*FKBP5* *MB-COMT* *BDNF* *NR3C1* *HTR3A* *COMT* *SLC6A4* *BDNF*
Hippocampal volume (n = 11)	*BDNF* *SLC6A4* *TESC* DNAm age (Horvath) Cluster of genes identified through EWAS (Hass et al., 2015)* OXTR DNAm age (Hannum) *FKBP5* *NR3C1-1F* *SLC6A4* *FKBP5*
Cortical volume (n = 2)	Cluster of genes identified through EWAS (Casey et al., 2017)* Cluster of genes identified through EWAS (Liu et al., 2015)*
Cortical thickness (n = 6)	*SLC6A4* *BDNF* *FKBP5* *FMR1* *SKA2* Independent component of DNAm (Freytag et al., 2017)
PFC (volume/thickness) (n = 2)	*OXTR* *SKA2*

*See Table 3, Table 4, Table 5, Table 6, Table 7, Table 8, Table 9 for list of genes included in clusters identified by EWAS.

Six studies tested an association between *SLC6A4* methylation and amygdala reactivity. Four out of five studies that reported associations between *SLC6A4* methylation and the emotional face processing task assessed by fMRI in response to threat stimuli found positive associations with amygdala reactivity and connectivity (Ismaylova et al., 2018; Nikolova et al., 2014; Schneider et al., 2018; Swartz et al., 2017). One study investigated resting state functional connectivity and reported a positive association between *SLC6A4* methylation and amygdala coupling with the salience network (Muehlhan et al., 2015). However, in a further study that investigated *SLC6A4* methylation and brain activity in the context of both resting state connectivity and the emotional face processing task, there were no significant associations reported with regard to the amygdala either with regards to whole brain analysis or amygdala region-of-interest analysis (Ismaylova et al., 2017). The sixth study that reported an association between *SLC6A4* and amygdala connectivity was also task-based fMRI, and used a visual emotional attention shifting paradigm (Frodl et al., 2015). These papers assessed DNAm within the promoter region of the *SLC6A4* gene, with the exception of Schneider et al., 2018, where a retrotransposon element, AluJb, was studied.

Two studies reported significant associations between *OXTR* methylation and amygdala connectivity (Puglia et al., 2015; Ziegler et al., 2015). Each study used a different fMRI task paradigm: the first used a task employing words involving social phobia relevant verbal stimuli in a cohort of patients with social anxiety disorder (Ziegler et al., 2015); and the second used an emotional face- matching block-design task in a group of healthy study participants (Puglia et al., 2015). Both studies measured DNAm in blood but each targeted a different region of the *OXTR* gene in bisulfite sequencing. Ziegler et al., 2015 targeted exon 3 of the *OXTR* gene due to previous work demonstrating an association between methylation in this region and social cognition (Unternaehrer et al., 2012; Ziegler et al., 2015). Puglia et al., 2015 targeted a single CpG site at position -934 relative to the transcription start site, where methylation had previously been associated with autism spectrum disorder (Gregory et al., 2009; Puglia et al., 2015). The directions of associations reported in these two studies were mixed, and may be explained by the variations in study design and participant characteristics.

Two studies reported significant associations between *BDNF* methylation and PFC functional connectivity (Moser et al., 2015; Ursini et al., 2016). Moser et al. performed DNAm-MRI analysis in a combined group of analysis of study participants with PTSD, sub-threshold PTSD and healthy control women, and assessed neural response to a Modified Crowell Procedure (Moser et al., 2015). The second study reported *BDNF* DNAm associations with PFC activity during a working memory task in a healthy group of study participants (Ursini et al., 2016). Both of these studies measured DNAm at different regions of the *BDNF* gene. Ursini et al., 2016 measured *BDNF* DNAm in blood at a region containing the rs6265 SNP, which lies in a coding region of the gene, which may interact with environmental factors to modulate risk for schizophrenia, while Moser et al., 2015 measured *BDNF* promoter DNAm in saliva, where methylation has been associated with trauma associated psychiatric disease in children (Moser et al., 2015; Ursini et al., 2016). The directions of associations reported in these studies were mixed.

Two studies reported associations between *FKBP5* methylation and hippocampal volume (Klengel et al., 2013; Resmini et al., 2016). In study participants with childhood trauma related PTSD there was a negative correlation between *FKBP5* methylation in blood and right hippocampal head volume (Klengel et al., 2013). In patients with Cushing’s syndrome there was a positive association between *FKBP5* methylation in blood and bilateral hippocampal volume (Resmini et al., 2016). Both of these studies measured DNAm at glucocorticoid responsive regions of the *FKBP5* gene, but report significant associations in different regions. Klengel et al., 2013 reported significant associations with intron 7, while Resmini et al., 2016 reports a significant association with a CpG site within intron 2 (Klengel et al., 2013; Resmini et al., 2016).

Two studies reported associations between *COMT* DNAm and PFC activation during working memory tasks (Ursini et al., 2011; Walton et al., 2014). The two studies assessed brain activity during different working memory tasks: the *n*-back task (Ursini et al., 2011); and the Sternberg Item Recognition task (Walton et al., 2014). The *COMT* gene encodes two isoforms of the catechol-O-methyltransferase enzyme: one soluble and one membrane bound, each with their own promoters, with the membrane bound isoform being the form most commonly found in the brain. Positive associations between *MB-COMT* promoter DNAm in blood and activity in the left dlPFC and vlPFC (Broca’s area 45) during the Sternberg Item Recognition task were found in a group of participants including healthy controls and schizophrenia patients (Walton et al., 2014). In the second study of healthy participants significant negative associations were reported between methylation at *COMT* rs4680 SNP in blood and activity in the bilateral PFC (BA 45/14 and BA47) during the *n*-back task in participants homozygous for the Val/Val allele at the rs4680 *COMT* SNP (Ursini et al., 2011). The difference in direction of associations reported by these two studies may be explained by the variations in study design and participant characteristics.

### 3.4 Associations between DNAm and MRI features categorised by phenotype

#### 3.4.1 Neurodevelopment and neurodevelopmental disorders

Table 3 summarises eight studies that focused on neurodevelopment: typical development; the impact of early life stress on young adulthood; *BDNF* polymorphism in early life; ADHD; preterm brain injury; hippocampal sclerosis in mesial temporal lobe epilepsy; birthweight discordance between twins and its impact on cortical anatomy. Four of the eight studies included a comparison group, and four were case series. Two studies report data from the Growing up towards Healthy Outcomes in Singapore (GUSTO) cohort study (Chen et al., 2015; Guillaume et al., 2018).

**Table 3:** Neurodevelopment and neurodevelopmental disorders.

Author, Country	Sample characteristics • Condition • Cases (male); controls (male) • Age	• Epigenome-wide/candidate, platform • Tissue sampled • Correction for cell sub-type distribution • Consideration of genotype	• MRI field strength • Modality • Image feature(s) • Analysis method	Main Findings
Chen et al., 2015 Singapore †	• *BDNF* Val66 Met polymorphism in GUSTO study • 237 cases (133 M) • Mean gestational age 38.7 weeks	• Epigenome-wide: Illumina 450 array • Umbilical cord blood • Cell type composition: NR • Consideration of genotype: yes	• 1.5 T • Structural, T2 • Volume of total brain and 18 ROIs • MRF model segmentation	• 19 % of total variable CpGs were significantly associated with the volume of the left hippocampus for Val/Val neonates. • 5% of total variable CpGs were associated with left hippocampal volume in the Met/Val genotype group • 3% of total variable CpGs were significant in the Met/Met genotype group.

Park et al., 2015 South Korea	• ADHD • 102 participants (77 M) • Mean age 8.9 years	• Candidate: *SLC6A4* - promoter (185bp region) • Blood • Cell type composition: NR • Consideration of genotype: yes	• 3 T • Structural, T1 • Cortical thickness • CLASP algorithm (Kim et al., 2005)	• Negative association between DNAm of *SLC6A4* CpGs 5-8 in the promoter region and cortical thickness in the right STG, middle occipital gyrus, lingual gyrus, inferior occipital gyrus, precentral gyrus, MTG, cuneus, and cingulate gyrus.

Sparrow et al., 2016 United Kingdom	• Preterm birth • 36 preterm infants (<32 weeks’ gestation); 36 term controls • Mean age at assessment: 39.7 weeks for controls and; 40.0 weeks for cases	• Epigenome-wide: Illumina 450 array • Saliva • Cell type composition: no • Consideration of genotype: yes	• 3 T • Diffusion • Tract shape in 8 major tracts. • Probabilistic Neighbourhood Tractography (PNT)	• 95 % of the variance in the preterm methylome was explained by 23 principal components; the 6^th^ principal component explained 2.9 % of the variance of DNAm data in the preterm group and was associated with right CST topology.

Casey et al., 2017 Canada	• Cortical anatomy in monozygotic twin cohort • 52 twin pairs (22 M) • Mean age 15.7 years	• Epigenome-wide: Illumina 450 array • Saliva • Cell type composition: yes • Consideration of genotype: NR	• 3 T • Structural, T1 • Cortical thickness, surface area, volume • CIVET automated analysis pipeline	• 23 probes mediate the relationship between birthweight discordance and cortical volume, including annotated probes found in 13 genes: *PRPF3, RELN, EXOC2, LEFTY2, CAST, SNK1G2, TRIM5, ABR, TNF, C6orf10, FREM2, GABPA* and *EFNA5*; 15 probes mediate the relationship between birthweight discordance and cortical surface area, including annotated probes found in 9 genes: *TMCO3, TRIM15, KDM2A, EPDR1, PARP14, PBLD, RELN, ACACA* and *CASZ1.* • No probes significantly mediated the relationship between birthweight discordance and cortical thickness discordance.

Long et al., 2017 China	• HS in MTLE • Cases n = 30 (18 M); controls n = 30 (18 M) • Cases mean 25.3 years; controls mean 28.2 years	• Epigenome-wide: Illumina 450 array • Blood • Cell type composition: NR • Consideration of genotype: NR	• Image acquisition: NR • Hippocampal sclerosis on conventional MRI	Subgroup analysis: MTLE patients with (n = 9) or without HS (n = 14).
				• From top 10 differentially methylated probes: DNAm in *SLC12A7, SRC*, *PLIN5* had a positive association with HS; DNAm in *PCNT, CEACAM21, PCGF3, BAT2* displayed a negative association with HS. • Genes from top 10 positively associated with HS: *RASAL3*, *PFDN6:WDR46*, *UBN1:GLYR1*, *TRIM49*, *ACVR1B*. • Genes from the top 10 negatively associated with HS: *SPIRE2*, *NKD1*, *TNFRSF11B*

Harms et al., 2017 United States	• Early life stress exposure on adult outcome • 33 participants (15 M) • Mean age 20.5 years	• Candidate: *FKBP5* - CpG sites in intron 7, 5, and 2 • Saliva • Cell type composition: NR • Consideration of genotype: NR	• 3 T • fMRI task: go-no go task • dlPFC activation • Analysis of Functional NeuroImages	• Negative association between DNAm of intron 5 of FKBP5 and activation in the dlPFC in the go no-go task. • CpG8 of intron 5 mediates the relationship between childhood stress and dlPFC activation.

Chen et al., 2018 United States	• ADHD • 15 monozygotic twin pairs (12 M), discordant for ADHD • Mean 9.7 years	• Epigenome-wide: Illumina 450 array • Blood • Cell type composition: yes • Consideration of genotype: yes	• 1.5 T • Structural, T1 • Cerebral cortex, cerebellum, caudate, putamen, thalamus volumes • FreeSurfer	Subset of 14 MZ twins:
				Of the 67 genes with intrapair probe DNAm which associated with volume discordance in the cerebellum, 49 were negatively associated. Of the 48 genes associated with the striatum, 38 were negatively associated, while of the 78 associated with the thalamus 44 were negatively associated.

Guillaume et al., 2018 Singapore †	• GUSTO birth cohort • 114 participants • 7-10 days post birth	• Epigenome-wide: Illumina 450 array • Umbilical cord blood • Cell type composition: yes • Consideration of genotype: NR	• 1.5 T, • Diffusion • FA • Voxel based comparison of FA maps	EWAS a significant association between probe cg19641625 in the *SMOC2* gene locus, with FA in the left vlThalamus.

^†^ Overlapping cohorts (GUSTO study).

Two studies used a candidate gene approach and both reported positive associations, but between different MRI features and different genes: the first between DNAm at the *SLC6A4* promoter and cortical thickness in an uncontrolled case series of 102 children with ADHD (Park et al., 2015); and the second between DNAm at *FKBP5* introns and dlPFC activation during the go no-go task in a case series of young adults with a history of early life stress exposure (Harms et al., 2017). The remaining six studies used the Illumina 450k array to measure DNAm, and all reported significant associations between DNAm at multiple loci and features including fractional anisotropy (FA), sub-cortical ROI volumes, cortical thickness, volume and surface area (Casey et al., 2017; Chen et al., 2015, 2018; Guillaume et al., 2018; Long et al., 2017; Sparrow et al., 2016). Two studies reported associations between DNAm at *SMOC2* and MRI brain imaging features (Chen et al., 2018; Guillaume et al., 2018). Guillaume et al., 2018 performed an EWAS in neonates and report an association between DNAm at this gene and FA in the left ventrolateral thalamus (Guillaume et al., 2018). Chen et al., 2018 reported differential methylation and discordance in twin pairs discordant for ADHD diagnosis, and identified six probes associated with the *SMOC2* gene which were differentially methylated and were associated with differences in striatum (among 48 probes that were differentially methylated with regard to striatum volume) (Chen et al., 2018). *SMOC2* encodes SPARC-related modular calcium binding protein 2.

Two studies reported an association between neonatal water diffusion properties and DNAm (Guillaume et al., 2018; Sparrow et al., 2016). In a case control study of preterm infants and term born controls principal component analysis of saliva DNAm identified a component that was associated with shape of the right corticospinal tract (Sparrow et al., 2016); and in neonates from the GUSTO cohort study there was an association between umbilical cord blood DNAm at the *SMOC2* gene and FA in the left ventrolateral thalamus (Guillaume et al., 2018).

#### 3.4.2 Major depressive disorder (MDD) and suicidality

Table 4 describes 10 studies of MDD and 1 of suicidality. Seven studies had overlapping participants: five have some overlapping participants from an outpatient psychiatric clinic of Korea University Anam Hospital and community controls (Choi et al., 2015; Han et al., 2017a, b; Na et al., 2016; Won et al., 2016); and two other studies had overlapping participants (Booij et al., 2015; Frodl et al., 2015). Nine studies included a comparator group and 2 were case series of individuals with familial risk for MDD.

**Table 4:** Major depressive disorder and suicidality.

Author, Country	Sample characteristics • Condition • Cases (male); controls (male) • Age	• Epigenome-wide/candidate, platform • Tissue sampled • Correction for cell sub-type distribution • Consideration of genotype	• MRI field strength • Modality • Image feature(s) • Analysis method	Main Findings
Choi et al., 2015 South Korea †	• MDD • Cases, n = 60 (13 M); controls, n = 53 (17 M) • Cases, mean 41.9 years; controls, mean 41.2 years	• Candidate: *BDNF-* 4 CpGs in the promoter region • Blood • Cell type composition: NR • Consideration of genotype: NR	• 3 T • Diffusion • FA/RD/AxD of 7 white matter tracts • Tract Based Spatial Statistics (TBSS)	• Cases: negative association between DNAm of CpG4 and FA of the right ACR, AxD of right ACR is negatively associated with *BDNF* DNAm. RD was not significantly associated with *BDNF* DNAm. • Controls: no significant positive associations.

Booij et al., 2015 Republic of Ireland ‡	• MDD • Cases, n = 33 (10 M); controls, n = 36 (15 M) • Cases, mean 40.3 years; controls, mean 35.3 years	• Candidate: *SLC6A4-* CpGs 5-15 found 214–625 bp upstream of the gene promoter • Blood • Cell type composition: NR • Consideration of genotype: yes	• 3 T • Structural, T1 • Whole hippocampal and sub-hippocampal volumes • FreeSurfer	• Whole group: negative predictive value of hippocampal volume and sub-volumes (CA2/3 and DG, and CA1) and *SLC6A4* DNAm (average and site specific – CpG 5&6, CpG 11&12)

Frodl et al., 2015 Republic of Ireland ‡	• MDD • Cases, n = 25(7 M); controls, n = 35(13 M) • Cases, mean 41.6 years; controls, mean 35.6	• Candidate: *SLC6A4-*promoter • Blood • Cell type composition: NR • Consideration of genotype: NR	• 3 T • fMRI task: Visual emotional attention shifting • Whole brain • SPM8	• Whole group: when shifting from emotional valence to geometric properties there is a negative association between DNAm and activation in the right Rolandic operculum and insula, right superior temporal lobe, and pons; when shifting from negative to neutral emotional valence, there is positive association between DNAm and activation in the left anterior insula and inferior frontal operculum • Controls: When looking at emotionally negative stimuli versus neutral stimuli, there was a positive association between DNAm and more activation in the bilateral hippocampus, left inferior operculum, fusiform gyrus and amygdala regions.

Na et al., 2016 South Korea †	• MDD • Cases, n = 65 (11 M); controls, n = 65 (15 M) • Cases, mean 42.5 years; controls mean 40.3 years	• Candidate: *BDNF* - 4 CpGs in the promoter region • Blood • Cell type composition: NR • Consideration of genotype: yes	• 3 T • Structural, T1 • Cortical thickness • FreeSurfer	• Cases: negative association between CpG2/4 and cortical thickness in the right rostral middle frontal, mOFC cortical thicknesses and left lingual, STG and SFG cortices. Negative association between CpG2 and cortical thickness in the right IT and pericalcarine and left rostral middle frontal cortices. Negative association between CpG4 and cortical thickness in the right cuneus, precuneus and post central cortices and left frontal pole cortices. • Controls: no significant associations found.

Won et al., 2016 South Korea †	• MDD • Cases, n = 35 (10 M); controls, n = 49 (15 M) • Cases, mean 40.3 years; controls mean 41.1 years	• Candidate: *SLC6A4-*5 CpGs in the promoter • Blood • Cell type composition: NR • Consideration of genotype NR	• 3 T • Diffusion • FA/MD/AxD/RD • TBSS	• Cases: negative association between *SLC6A4* DNAm and FA/AxD in the BCC (medication naïve). • Controls: negative association between *SLC6A4* DNAm and GCC FA, and positive association with RD. • Associations between BCC FA/AxD and CpG3 DNAm are higher in the cases compared to controls.

Han et al., 2017a South Korea †	• MDD • Cases, n = 114 (24 M); controls, n = 88 (27 M) • Cases, mean 43.5 years; controls, mean 39.9 years	• Candidate: *FKBP5* - intron 7 rs1360780, 2 CpGs investigated • Blood • Cell type composition: NR • Consideration of genotype: yes	• 3 T • Structural, T1 • GM volumes/cortical thickness of 14 cortical/subcortical regions • FreeSurfer	• *FKBP5* C homozygote group (patients and controls) (n = 118): positive association between DNAm of CpG1 and cortical thickness in the right transverse frontopolar gyrus.

Kim et al., 2017 South Korea	• Suicidality • Cases (suicide attempters), n = 14 (1 M), controls, n = 22 (9 M) • Cases, mean 31.9 years, controls, 33.6 years	• Candidate: *CACNA1C* - 11 CpG sites in DMR in a transcription factor binding site • Blood • Cell type composition: NR • Consideration of genotype: NR	• 3 T • fMRI task: participants shown pictures of facial emotion and objects relating to suicidal means • Activation in 4 left hemisphere regions • SPM5	• Cases: in knife vs neutral landscape contrast positive association between CpG4/6 DNAm and brain activation in the left thalamus, MFG, IFG. • Controls: positive association between CpG6 DNAm and activation in the left MFG and IFG.

Han et al., 2017b South Korea †	• MDD • Cases, n = 84 (13 M); controls, n = 61(17 M) • Cases, mean 43.4 years; controls, mean 38.6 years	• Candidate: *TESC-*rs7294919 locus • Blood • Cell type composition: NR • Consideration of genotype: yes	• 3 T • Diffusion • Structural • Diffusion parameters of the PHC • Hippocampal subfield volumes • Global PNT	• Cases: negative association of FA and positive association of RD with CpG3 DNAm in right PHC. • Controls: no significant associations.

Swartz et al., 2017 United States	• Family history of depression • Cases n = 94 (51 M); controls n = 89(46 M); • Wave 1, mean 13.5 years; wave 2, mean 15.6 years	• Candidate: *SLC6A4* - 20 CpG sites in promoter region • Blood • Cell type composition: NR • Consideration of genotype: yes	• 3 T • fMRI task: emotional face processing • Amygdala reactivity • SPM8	• Subgroup (n = 87), longitudinal analysis: *SLC6A4* promoter DNAm increases over time is associated with increases in left centromedial amygdala reactivity when participants viewed faces with fearful expressions in contrast to geometric shapes.

Davis et al., 2017 United States	• High/low familial MDD risk among females • High risk, n = 24; low risk, n = 22 • High risk, mean 12.9 years; low risk, mean 12.1 years	• Epigenome-wide: Illumina EPIC, Horvath DNAm age • Saliva • Cell type composition: yes • Correction of genotype: NR	• 1.5 T/3 T • Structural, T1 • Hippocampal and amygdala volumes • FreeSurfer	Whole group analysis:
				• Negative associations between DNAm age residual and left hippocampal volume. • Accelerated DNAm age mediates the effect of cortisol on left hippocampal volume.

Schneider et al., 2018 Germany	• MDD • Cases, n = 137 (57 M); controls, n = 189 (83 M) • Cases, mean 37.4 years; controls, mean 35.6 years	• Candidate: *SLC6A4* - AluJb element • Blood • Cell type composition: NR • Consideration of genotype: yes	• 3 T • fMRI task: emotional face processing (threat/fearful faces) • Amygdala reactivity • SPM8	• Whole group analysis: Significant interaction of amygdala activity with DNAm and diagnosis. • Cases: positive association between AluJb DNAm and activity in the right amygdala. No effect of DNAm on bilateral amygdala reactivity • Controls: no significant associations

^†^ Overlapping cohorts.

^‡^ Overlapping cohorts.

Ten of eleven studies used a candidate gene approach based on blood samples and one carried out EWAS on salivary DNA. Five candidate genes were studied in the context of MDD: *SLC6A4* (n = 5), *BDNF* (n = 2), *TESC* (n = 1), *FKBP5* (n = 1) and *CACNA1C* (n = 1). The single EWAS study reported an association between DNAm age and hippocampal volume, noting that DNAm mediated the effect of cortisol on hippocampal volume (Davis et al., 2017).

All four fMRI studies were task based. Two used the emotional face processing paradigm to investigate amygdala reactivity in relation to the *SLC6A4*AluJb element or its promoter, and both report significant correlations between activation and *SLC6A4* DNAm among cases (Schneider et al., 2018; Swartz et al., 2017). Another study reported a correlation between DNAm at the *SLC6A4* and limbic system activation during a visual emotional attention shifting task in an analysis which pooled MDD cases and controls (Frodl et al., 2015). Together these studies suggest that *SLC6A4* methylation positively correlates with limbic system reactivity in response to threat or negative emotional stimuli. However, these studies measured and reported *SLC6A4* methylation at different sites, and analysed them differently. For example, Frodl et al. used a summary mean measure calculated from 11 CpG sites in the promoter, Swartz et al. calculated a residualised change score from 20 CpG sites, and Schneider et al. calculated mean methylation at the AluJb element of the promoter from 6 CpG sites (Frodl et al., 2015; Schneider et al., 2018; Swartz et al., 2017). The fourth fMRI study investigated brain responses to images that were associated with suicidal means in relation to DNAm in the *CACNA1C* transcription factor binding site, and reported differential brain activation in association with DNAm at two CpGs (Kim et al., 2017).

#### 3.4.3 Alcohol use disorder

Table 5 summarises 4 studies of alcohol use disorder, all of which investigated DNAm in candidate genes in relation to task-based fMRI. Two were case-control study designs and two were uncontrolled case series. Three candidate genes were assessed: *DRD2*, *SLC6A3/DAT* and *PPM1G*. Three different tasks were used: testing impulse control, reactivity to alcohol cues and processing of reward/punishment cues. Three of these studies measured DNAm in blood, and one in saliva. Three of the four studies reported positive correlations between functional activation in limbic system structures and DNAm among participants with alcohol use disorder (Bidwell et al., 2018; Ruggeri et al., 2015; Wiers et al., 2015). The remaining study found no significant correlations in the alcohol dependent cases, but did find negative correlations in functional activation in the nucleus accumbens and methylation at the *SLC6A3* promoter in the context of a monetary incentive delay task (Muench et al., 2018*)*.

**Table 5:** Alcohol use disorder.

Author, Country	Sample characteristics • Condition • Cases (male); controls (male) • Age	• Epigenome-wide/candidate, platform • Tissue sampled • Correction for cell sub-type distribution • Consideration of genotype	• MRI field strength • Modality • Image feature(s) • Analysis method	Main Findings
Ruggeri et al., 2015 United Kingdom	• Alcohol exposure (IMAGEN cohort) • n = 499(222 M) • Mean age 14 years	• Candidate: *PPM1G* - 3’ UTR • Blood • Cell type composition: NR • Consideration of genotype: yes	• 3 T • fMRI task: stop signal task - impulse control • BOLD responses in 2 ROIs • MarsBaR (SPM8)	Subset of whole sample (n = 393)
				• Positive association between *PPM1G* DNAm and BOLD response in the right subthalamic nucleus in the stop success versus go success contrast • No association with right IFG activation

Wiers et al., 2015 United States	• Alcohol dependence • Cases n = 38 (M); controls, n = 17 (M) • Cases, mean age 44.4 years; controls, mean 42.7 years	• Candidate: *SLC6A3/DAT -* 12 CpG sites in promoter region (-1037 to -829) • Blood • Cell type composition: NR • Consideration of genotype: NR	• 3 T • fMRI task: cue reactivity task, alcohol elicited response • Reactivity in 4 ROIs • MarsBaR (SPM8)	• Cases: positive association between alcohol elicited amygdala reactivity (in contrast to soft drink) and *DAT* DNAm found only in cases with low depression scores (n = 29)

Bidwell et al., 2018 United States	• Alcohol dependence • 383 (238 M) • Mean age 30.1 years (range 21–56)	• Candidate: *DRD2* - 6 CpGs in promoter region (-126 to -189) • Saliva • Cell type composition: NR • Consideration of genotype: NR	• 3 T • fMRI task: cue reactivity task, alcohol elicited response • BOLD contrasts of 10 ROIs and WB analysis • SPM8	• ROI analysis: Positive association between DRD2 DNAm and alcohol elicited activation in striatum: right NAcc, bilateral putamen and bilateral caudate. • WB analysis: positive associations between DRD2 DNAm and alcohol elicited response in: left superior temporal, middle temporal, insula, postcentral, supramarginal, putamen regions.

Muench et al., 2018 United States	• Alcohol dependence • Cases, n = 45(35 M); controls, n = 45 (22 M) • Cases: mean 43.3 years; controls: 36.2 years	• Candidate: *SLC6A3/DAT-*48 CpGs • Blood • Cell type composition: NR • Consideration of genotype: NR	• 3 T • fMRI task: Monetary incentive delay task • BOLD responses in bilateral NAcc • AFNI	• Cases: no significant associations • Controls: negative association between SLC6A3 promoter DNAm and NAcc activation during the anticipation of both high and low monetary loss. The effects were driven by 2 CpGs located at positions -1,001 and -993 from the TSS in the promoter of *SLC6A3*.

#### 3.4.4 Schizophrenia and psychosis

Table 6 summarises seven studies of schizophrenia and psychosis, all of which included a comparator group. Six of the included studies use data from participants in the Mind Clinical Imaging Consortium (MCIC) all of whom have MRI, DNAm and SNP data available (Deng et al., 2016; Hass et al., 2015; Hu et al., 2018; Liu et al., 2015; Walton et al., 2014; Wang et al., 2017).

**Table 6:** Schizophrenia and psychosis.

Author, Country	Sample characteristics • Condition • Cases (male); controls (male) • Age	• Epigenome-wide/candidate, platform • Tissue sampled • Correction for cell sub-type distribution • Consideration of genotype	• MRI field strength • Modality • Image feature(s) • Analysis method	Main Findings
Walton et al., 2014 United States †	• Schizophrenia or schizophreniform disorder • Cases, n = 82(62 M); controls, n = 102(62 M) • Cases, mean 33.8 years; controls 32.7 years	• Candidate: *MB-COMT* - 864bp upstream from TSS of membrane bound isoform of *COMT* • Blood • Cell type composition: NR • Consideration of genotype: yes	• 1.5 T and 3 T • fMRI task: Sternberg item recognition paradigm - working memory task. • BOLD response in dlPFC and exploratory whole brain analysis • fMRIB (FSL)	Whole group:
				• ROI analysis: positive association between *MB-COMT* promoter DNAm and left dlPFC activation; • Whole brain analysis: positive association between *MB-COMT* promoter methylation and left dlPFC and vlPFC (BA45), left premotor and primary sensory cortex. No effect in R dlPFC. No effect of diagnosis.

Liu et al., 2015 United States †	• Schizophrenia, schizophreniform or schizoaffective disorder • Cases, n = 94; controls, n = 106 • Cases, mean 34.7 years; controls, mean 32.5 years	• Epigenome-wide: Illumina Infinium HumanMethylation27 Array • Blood • Cell type composition: NR • Consideration of genotype: NR	• 1.5 T and 3 T • Structural, T1 • Volumes of cortical and subcortical regions • FreeSurfer	• Negative association between the 7^th^ DNAm component and the 1^st^ brain volume component:
				Brain volume component - left cerebellar cortex, right cerebellar cortex
				Methylation component - *C1orf65, MCCC1, EPHA3, CDX1, RAET1L, LR8, STMN2, GATA4, NALP6, KCNK10, ADRA1D, NPDC1, MCHR1*

Hass et al., 2015 United States †	• Schizophrenia • Cases, n = 103 (75 M); Controls, n = 111(71 M) • Cases: mean 34.6 years; controls, mean 32.1 years	• Epigenome-wide: Illumina Infinium HumanMethylation27 Array • Blood • Cell type composition: yes • Consideration of genotype: yes	• 1.5 T and 3 T • Structural, T1 • fMRI: Sternberg Item Recognition Paradigm - working memory task. • Hippocampal volume, STG thickness • Working memory load dependent neural activity (percentage BOLD signal change) in the dlPFC • FreeSurfer • fMRIB (FSL)	• Alterations in the DNAm state of genes within the hsa-miR-219a-5p targets are associated with hippocampal volume changes (has-miR-219a-5p target gene set: *KIAA0182, PKNOX1, TRAF7, CBFA2T3, EPHA4, MKNK2, DDAH1, ESR1, AKAP13, PHACTR2, SLC31A1, KBTBD8, ADD2, ERGIC1, RBM24, KIAA0240, NEK6, THRB, SCN5A, TGFBR2, FZD4, SEMA4G)* • STG thickness: miR-513 gene set sig enriched (NES = 1.7636) with an FDR q value of 0.2404 (based on positive association between DNAm data and phenotype) • None of the predicted miRNA target gene sets was significantly enriched in association with working-memory load-dependent neural activity in the dlPFC.

Deng et al., 2016 United States †	• Schizophrenia • Case, n = 80(60 M); Controls, n = 104 (66 M) • Cases, mean 34 years; controls, mean 32 years	• Epigenome-wide: Illumina Infinium HumanMethylation27 Array • Tissue sampled: blood • Cell type composition: NR • Consideration of genotype: NR	• NR • fMRI • NR • NR	• Small concordance between fMRI data and DNAm data. • Prediction accuracy of schizophrenia cases was improved by incorporating the two data types versus prediction models using one or the other.

Rubin et al., 2016 United States	• Schizophrenia and Bipolar disorder • Case, n = 167(75 M); controls, n = 75(37 M) • Age Schizophrenia: Women (n = 22): mean 39 years; Men (n = 35): mean 22 years Schizoaffective disorder: Women (n = 19) mean 37 years; Men (n = 15) mean 38 years Bipolar disorder: Women (n = 51) mean 36 years; Men (n = 25) mean 26 years Controls: Women (n = 38) mean 36 years; Men (n = 37) mean 39 years	• Candidate: *OXTR* - methylation measured at a site 934bp upstream of TSS • Blood • Cell type composition: NR • Consideration of genotype: NR	• 3 T • Structural, T1 • Volumes of prefrontal regions and temporal limbic regions • FreeSurfer	• Schizophrenia: positive association between OXTR DNAm and volumes of the left OFC, and right fusiform gyrus and negative association with the right hippocampus. • Bipolar disorder: positive association between DNAm and right parahippocampal gyrus volume. • Schizoaffective disorder (men): Negative association between *OXTR* DNAm and left MFG volume. • Controls: negative association between *OXTR* DNAm and left amygdala, right MFG and IFG volumes.

Wang et al., 2017 United States †	• Schizophrenia • Cases, n = 80(60 M); Controls, n = 104 (66 M) • Cases: mean 34 years; controls mean 32 years	• Epigenome-wide: Illumina Infinium HumanMethylation27 Array • Blood • Cell type composition: NR • Consideration of genotype: NR	• NR • fMRI • NR • NR	• Positive association between fMRI and DNAm modules in cases. • Associations in controls do not reach statistical significance. • DNAm module: *ANKRD15, ATP6V0A4, C1orf116, C1orf172, C21orf56, CCNA1, CREB3L3, FLJ11017, HOXA4, HTN3, KIF27, LDHD, PAGE5, PDIA2, RCBTB2, RPL26L1, TMEM100* • fMRI module: midcingulate area, parahippocampal gyrus, postcentral gyrus, supramarginal gyrus, angular gyrus, precuneus, superior temporal gyrus

Hu et al., 2018 United States †	• Schizophrenia • Cases, n = 79; controls, n = 104 • Age: NR	• Epigenome-wide: Illumina Infinium HumanMethylation27 Array • Blood • Cell type composition: NR • Consideration of genotype: NR	• Field Strength: NR • fMRI task: Sensorimotor task • NR • NR	• Positive association between DNAm and fMRI data • DNAm: 31 CpG sites that correspond to 30 genes • fMRI (7 ROIs): left inferior frontal gyrus (pars triangularis), left hippocampus, right inferior occipital gyrus, bilateral fusiform gyrus, right middle temporal gyrus, left inferior temporal gyrus

^†^ Mind Clinical Imaging Consortium.

The majority of these studies measured blood DNAm on the Illumina HumanMethylation27 array (n = 4), and one study carried out a candidate gene analysis on blood *MB-COMT* DNAm. Five of these used the Illumina HumanMethylation27 array, and two carried out bisulfite pyrosequencing of two genes: *OXTR* and *MB-COMT*. fMRI was the most commonly used modality (n = 5), followed by sMRI (n = 3). Of these, one study used both modalities. fMRI tasks included: a sensorimotor task and the Sternberg Item Recognition Paradigm for working memory (Hu et al., 2018; Walton et al., 2014). Two studies did not report the fMRI paradigm (Deng et al., 2016; Wang et al., 2017).

#### 3.4.5 Ageing, stroke, ataxia and neurodegeneration

Table 7 summarises eight studies that investigated aspects of ageing, including healthy ageing, Alzheimer’s disease, minor cognitive impairment, stroke, WMH burden and *C9orf72* expansion and Fragile X Associated Tremor Ataxia Syndrome (FXTAS).

**Table 7:** Ageing, stroke, ataxia and neurodegeneration.

Author, Country	Sample characteristics • Condition • Cases (male); controls (male) • Age	• Epigenome-wide/candidate, platform • Tissue sampled • Correction for cell sub-type distribution • Consideration of genotype	• MRI field strength • Modality • Image feature(s) • Analysis method	Main Findings
McMillan et al., 2015 United States	• *C9orf72* expansion • Cases, n = 20; Controls, n = 25 • Cases: mean 61.9 years; controls: mean 6138 years	• Candidate: *C9orf72* • Blood • Cell type composition: NR • Consideration of genotype: NR	• 3 T • Structural, T1 • Grey matter density • Randomise (FSL)	• *C9orf72* expansion group: positive association between *C9orf72* DNAm and grey matter density in the right hippocampus and thalamus and left premotor cortex. • Longitudinal analysis (n = 11): GM atrophy progresses more rapidly over time with decreased *C9orf72* DNAm in patients with *C9orf72* expansion

Shelton et al., 2016 Australia †	• Fragile X associated tremor ataxia syndrome (FXTAS) • Cases, n = 19 F; controls, n = 17F • Cases: mean 39.4 years; controls, mean 39.8 years	• Candidate: FMR1 - exon 1 CpG1 and CpG2; intron 1 CpG6/7, CpG8/9; CpG10-12 • Blood • Cell type composition: NR • Consideration of genotype: NR	• 3 T • Structural, T1 • Regional cortical thickness from bilateral MFG and SFG and inferior parietal gyrus • FreeSurfer	• Premutation group: positive associations between methylation of *FMR1* CpG6/7 or CpG1 or CpG10-12 and of cortical thickness in the left inferior parietal gyrus, middle frontal gyrus, and right middle frontal gyrus, superior frontal gyrus. Negative association between methylation in the intronic region with white matter hyperintensities. • Controls: negative associations between FMR1 DNAm at CpGs 2, 6/7, 10–12 and cortical thickness in frontal lobe regions: left middle frontal gyrus and right superior frontal gyrus

Zhou et al., 2016 China	• Cerebral infarction • Cases, n = 83(45 M); controls, n = 94(52 M) • Cases: mean 61.6 years; controls: mean 61.6 years	• Candidate: Extracellular *SOD* • Blood • Cell type composition: NR • Consideration of genotype: NR	• Field strength: NR • MR Angiography • Area of cerebral infarction; cerebral arteriosclerosis • Analysis: NR	• Dependence between large/small area of cerebral infarction and extent of extracellular *SOD* DNAm. • Negative but non-significant association between arteriosclerosis severity and extracellular *SOD* DNAm

Shinagawa et al., 2016 Japan	• Alzheimer’s/aMCI • Cases 58(27 M): AD, n = 30; aMCI n = 28 • Cases, mean 71.9 years	• Candidate: *NCAPH2/LMF2* promoter – representative CpG site • Blood • Cell type composition: NR • Consideration of genotype: NR	• 1.5 T • Structural, T1 • VBM Grey matter/ VSRAD Z-score (hippocampal atrophy grade) • SPM8, DARTEL/VSRAD	• Extent of grey matter atrophy non-significant for associations with NCAPH/LMF2 promoter DNAm. • Positive association between DNAm of *NCAPH2/LMF2* and severity of hippocampal atrophy.

Hodgson et al., 2017 United States	• Ageing • N = 376	• Epigenome-wide: Illumina 450 array, Horvath DNAm age • Blood • Cell type composition: yes • Consideration of genotype: yes	• 3 T • Diffusion • FA • TBSS	• Negative association of FA with epigenetic age acceleration.
	• Mean age 45.5 years			

Raina et al., 2017 United States	• Cerebral white matter hyperintensities • N = 713(257 M) • Mean age at MRI was 61.8 years; mean age at DNA sampling 59.3 years	• Epigenome-wide; Illumina 450k array, Hannum/Horvath DNAm age • Blood • Cell type composition: yes • Consideration of genotype: yes	• 1.5 T • Structural, T1/T2 • White matter hyperintensities • Non-automated counting	• Positive association between WMH burden and DNAm age acceleration: high WMH burden category was associated with DNAm age acceleration, whereas low WMH burden was not. • No individual probe reached significance in any analysis.

Shelton et al., 2017 Australia †	• FXTAS • Cases n = 20 F; controls, n = 20 F • Cases mean 40.1 years; controls mean 39.1 years	• Candidate: *FMR1* – exon 1 CpG1 and CpG2; intron 1 CpG6/7, CpG8/9; CpG10-12 • Blood • Cell type composition: NR • Consideration of genotype: NR	• 3 T • Diffusion • FA, MD in ROIs • TBSS (FSL)	• Premutation group: negative association between inferior and middle cerebellar peduncle’s MD and CpG1 • Control: negative association between BCC FA and CpG6/7

Chouliaras et al., 2018 United Kingdom	• N = 47(39 M) • Cognitively intact, mean 72.8 years; cognitively impaired, mean 72.3 years (s.d. 6.02)	• Epigenome-wide: Illumina 450k array, Horvath/Hannum DNAm age • Blood • Cell type composition: yes • Consideration of genotype: NR	• 3 T • Structural, T1/ • Diffusion • total brain, hippocampal, CSF, GM volumes, WMH • FA, MD • Voxel wise statistical analysis, BIANCA (WMH detection)/ FSL	• No associations were observed between the Horvath epigenetic clock and any of the imaging variables tested • Candidate gene testing did not find any associations that survived correction for multiple comparisons. • Negative association between global MD and Hannum age, and positive association between global FA and Hannum age.

^†^ Overlapping cohort.

Imaging modalities used were as follows: dMRI (n = 2), sMRI (n = 5) d/sMRI (n = 1). All studies used blood samples to measure DNAm. Five studies carried out candidate gene analysis and three measured DNAm in an epigenome-wide manner. The candidate genes studied were: *C9orf72* (n = 1), *NCAPH2/LMF2* (n = 1), *FMR1* (n = 2), *SOD* (n = 1). Two studies carried out a longitudinal analysis as well as a cross-sectional analysis (McMillan et al., 2015; Swartz et al., 2017). One of these showed, in a group of 11 *C9orf72* expansion patients, that there was more rapid grey matter atrophy in the right hippocampus, right thalamus and left middle frontal cortex where there was decreased methylation in the expansion region (McMillan et al., 2015). The other found in 87 study participants that increases in *SLC6A4* promoter methylation corresponded to increases in amygdala reactivity in response to threat related stimuli in task-based fMRI (Swartz et al., 2017). Two studies that investigated associations between *FMR1* methylation and brain imaging in women with FXTAS had overlapping participants (Shelton et al., 2017, 2016). The three EWAS studies investigated DNAm age in relation to its associations with brain imaging features (Chouliaras et al., 2018; Hodgson et al., 2017; Raina et al., 2017). Two of these three studies investigated dMRI: one found a negative association of FA with epigenetic age acceleration, while the other found a negative association between global mean diffusivity (MD) and Hannum age and a positive association between FA and Hannum age (Chouliaras et al., 2018; Hodgson et al., 2017). In an analysis with sMRI a positive association was found between WMH burden category and DNAm age acceleration (Raina et al., 2017).

#### 3.4.6 Post-traumatic stress disorder

Table 8 summarises seven studies that were carried out in participants with PTSD (Table 6). Several studies had overlapping participants. Three studies report associations in an overlapping group of participants: PTSD cases and non-PTSD controls, where the case population was mothers who have experienced interpersonal violence (IPV) (Moser et al., 2015; Schechter et al., 2017, 2015). Two further studies had overlapping participants – a cohort of trauma exposed veterans (Sadeh et al., 2016; Wolf et al., 2016). An additional group of veterans was also studied in relation to PTSD (McNerney et al., 2018). A further study recruited women who had experienced early life trauma to investigate associations between DNAm and sMRI features (Klengel et al., 2013). Five candidate genes were studied: *NR3C1* (n = 2), *SKA2* (n = 1), *BDNF* (n = 1), *FKBP5* (n = 1), *5HT3A* (n = 1). All three modalities were employed to investigate neuroimaging features: dMRI (n = 1), sMRI (n = 3), fMRI (n = 3). Overlap in study populations lay between two studies which measured DNAm using the Illumina 450k array (Sadeh et al., 2016; Wolf et al., 2016). Wolf et al. (2016) carried out analysis of DNAm age while Sadeh et al. (2016) carried out a candidate gene analysis of *SKA2* (Sadeh et al., 2016; Wolf et al., 2016). McNerney et al. (2018) measured DNAm at the *NR3C1* gene using bisulfite pyrosequencing (McNerney et al., 2018). All three of the articles investigating a population of mothers who have experienced IPV studied candidate genes: *NR3C1, BDNF* and *5HT3A* (Moser et al., 2015; Schechter et al., 2017, 2015). All three used fMRI, with two using the same task (Modified Crowell procedure), and one using a unique task video stimulus depicting neutral, menacing, and male-female prosocial interactions (Moser et al., 2015). The two studies that reported on IPV PTSD mothers in relation to the modified Crowell procedure showed positive associations between DNAm at *NR3C1* and *BDNF* and cluster activation in vmPFC OFC posterior cingulate cortex (Moser et al., 2015; Schechter et al., 2015).

**Table 8:** Post-traumatic stress disorder.

Author, Country	Sample characteristics • Condition • Cases (male); controls (male) • Age	• Epigenome-wide/candidate, platform • Tissue sampled • Correction for cell sub-type distribution • Consideration of genotype	• MRI field strength • Modality • Image feature(s) Analysis method	Main Findings
Klengel et al., 2013 United States	• Childhood trauma related PTSD • Cases n = 56(F) • Cases, mean age 28.5 mean years	• Candidate: *FKBP5* - 4 intronic regions, CpG island of TSS and a region upstream of the TSS • Blood • Cell type composition: NR • Consideration of genotype: yes	• 3 T • Structural, T1 • Amygdala/Hippocampal volume • Amygdala and hippocampal segmentation (Pruessner et al., 2000)	• Negative association between FKBP5 (mean at intron 7 bin 2) DNAm and right hippocampal head volume in a subset of the cohort (n = 34).

Moser et al., 2015 Switzerland †	• Inter-personal violence related PTSD • Controls, n = 20 F; Subthreshold PTSD, n = 8 F; IPV-PTSD diagnosis, n = 18 F • Age - (sample of 68 mothers with saliva): Mean 34.3 years (s.d. 5.8 years)	• Candidate: *BDNF* - mean methylation of 4 CpGs found in exon IV • Saliva • Cell type composition: NR • Consideration of genotype: NR	• 3 T • fMRI task: Modified Crowell Procedure • Contrast of brain activity when mothers watched their own and unfamiliar children during scenes of separation vs play • SPM8	Whole group analysis:
				• Positive associations between *BDNF* promoter mean methylation and DNAm and cluster activation in the following clusters: in left vmPFC, OFC, sgACC; right vmPFC, OFC; posterior cingulate cortex. • Negative associations between *BDNF* promoter mean methylation and cluster activation in the following clusters: right hippocampus, right parahippocampal gyrus, right fusiform gyrus; left precuneus; right precuneus; left cerebellum; right STG.

Schechter et al., 2015 Switzerland †	• Inter-personal violence related PTSD • Cases, n = 28; controls n = 17 • Cases, median 35.1 years; controls, median 35.5 years	• Candidate: *NR3C1* – mean methylation of 13 CpGs • Saliva • Cell type composition: NR • Consideration of genotype: NR	• 3 T • fMRI task: Modified Crowell Procedure • Batch programming based on SPM8	Whole group analysis:
				• Positive associations between DNAm of *NRC31* and cluster activation in vmPFC OFC, right gyrus rectus; left dlPFC; left dlPFC, left precentral gyrus; dmPFC; left precuneus, posterior cingulate cortex • Spearman’s correlations between each of the 13 CpG sites and neural activity in prefrontal cortex regions during separation vs. play did not reach statistical significance

Sadeh et al., 2016 United States ‡	• PTSD • Cases: a subset (n = 145) of cohort size n = 200(182 M) • Mean 31.8 years	• Candidate: *SKA2*– 3’ UTR. (Illumina probe cg13989295) • Illumina 450 array • Blood • Consideration of genotype: yes	• 3 T • Structural, T1 • Cortical thicknesses • FreeSurfer	• Negative associations between *SKA2* DNAm and cortical thickness in prefrontal regions: left frontal pole, SFG and rostral middle frontal gyrus; right frontal pole, SFG, and right OFC/IFG pars orbitalis and rostral MFG. • Mediation was significant for right OFC and rostral MFG cluster, and for left prefrontal cluster. Mediation effect not significant for the right frontal pole/SFG cluster, though direction of association was consistent.

Wolf et al., 2016 United States ‡	• PTSD • Cases, n = 281(247 M) • Cases, mean age 31.9 years	• Epigenome-wide: Illumina 450 array, Horvath/Hannum DNAm age Blood • Cell type composition: yes • Consideration of genotype: yes	• 3 T • Diffusion • FA/RD/MD/AxD in right frontal cortex, left frontal cortex, genu of corpus callosum • FreeSurfer/TBSS	Subset (n = 241):
				• Accelerated Hannum DNAm age associated with decreased FA, increased RD and MD in genu of corpus callosum • Positive association between DNAm age acceleration and RD and MD in genu of corpus callosum • Negative association between DNAn age acceleration and FA in the genu of corpus callosum. • No associations found with Horvath DNAm age.

Schechter et al., 2017 Switzerland †	• Inter-personal violence related PTSD • Cases, n = 18 F; controls, n = 17F • Cases, mean age 33.5 years; controls, mean 35.6 years	• Candidate: serotonin receptor 3A methylation (*HTR3A*) – 7 CpG sites in the promoter region • Saliva • Cell type composition: NR • Consideration of genotype: NR	• Field strength: NR • fMRI task: video stimuli depicting neutral, menacing, and male-female prosocial interactions. • Neural activity in maternal brain regions association with emotion regulation • Batch programming based on SPM8	• Negative association between DNAm at CpG 2 III site and neural activity in response to menacing vs prosocial stimuli: dorsomedial PFC, dACC, bilateral dorsolateral prefrontal gyrus, left prefrontal gyrus, left temporal pole, left STG, left MTG; posterior cingulate cortex; left cuneus, V1; cerebellum; right brainstem. • Negative association between DNAm at *5HT3A* CpG 2 III site and neural activity in response to menacing vs neutral stimuli: dmPFC, left dlPFC, left temporal pole, left middle temporal gyrus.

Mcnerney et al., 2018 United States	• Veterans risk of meeting PTSD diagnostic criteria • Cases, n = 67 (59 M) • Cases, mean 46 years	• Candidate: *NR3C1-1F* – promoter binding sites for transcription factor NGF1A • Saliva • Cell type composition: NR • Consideration of genotype: NR	• 3 T • Structural, FSPGR • Intracranial volume and hippocampal volume • FreeSurfer	• Positive associations between hippocampal volume and DNAm of *NR3C1* gene in low scorers on the PTSD checklist (Civilian version 4; PCL-C), unlikely to meet PTSD diagnostic criteria). • Associations were not significant for those who scored highly on the PCL-C.

^†^ Overlapping cohort.

^‡^ Overlapping cohort.

#### 3.4.7 Miscellaneous: healthy adults, socio-emotional processing, Cushing’s syndrome

Two of the included studies in this section investigated patient populations, and the remainder reported healthy participants. One case-control study investigated DNAm-MRI in Cushing’s Syndrome patients, and another case series study investigated social anxiety disorder (Resmini et al., 2016; Ziegler et al., 2015). Both studies discovered significant correlations between DNAm and neuroimaging findings. Fourteen studies in this section adopt a candidate gene analysis approach, with 1 being epigenome-wide (Table 9). The candidate genes studied were: *COMT* (n = 1)*, OXTR* (n = 3)*, NR3C1* (n = 1)*, SLC6A4* (n = 5)*, OXT* (n = 1)*, FKBP5* (n = 1)*, KLF13* (n = 1), *BDNF* (n = 1). DNAm was measured in the following tissues: saliva (n = 3); blood (n = 10); blood/saliva/buccal (n = 1); saliva and blood (n = 1). Two MRI modalities are represented in this section: sMRI (n = 3), and fMRI (n = 10), and two studies used both modalities (s/fMRI (n = 2). fMRI tasks included: N back task, social phobia associated verbal stimuli, emotional perspective, emotional attribution, social perception, emotional face processing, responses to high calorie versus low calorie foods.

**Table 9:** Miscellaneous: healthy adults, emotion processing, social anxiety disorder, Cushing’s syndrome.

Author, Country	Sample characteristics • Condition • Cases (male); controls (male) • Age	• Epigenome-wide/candidate, platform • Tissue sampled • Correction for cell sub-type distribution • Consideration of genotype	• MRI field strength • Modality • Image feature(s) • Analysis method	Main Findings
Ursini et al., 2011 Italy	• Healthy participants • Cases, n = 84(32 M) • Cases, mean age 25.9 years	• Candidate: *COMT-* rs4680 (C2) and gene promoter (3 CpGs in the promoter) • Peripheral blood mononuclear cells • Cell type composition: NR • Consideration of genotype: yes	• 3 T • fMRI task: N-back task–working memory • BOLD responses in the bilateral prefrontal cortex • SPM5	Val/Val genotype:
				• Negative association between C2 DNAm and bilateral PFC (BA 45/13 and BA47) activity during task • Increased stress and lower C2 DNAm related to greater activity in bilateral PFC (BA10/47; BA44/45) during working memory task • No significant relationship was found between DNAm of sites in the COMT promoter or in LINE-1 and working memory related PFC activity in any other genotype group or in the whole sample.

Jack et al., 2012 United States	• Healthy participants • Cases, n = 42(23 M) • Cases, mean age 21.9 years	• Candidate: *OXTR*- coding strand including site -934 • Blood (mononuclear cells) • Cell type composition: NR • Consideration of genotype: NR	• 3 T • fMRI task: social perception task (animacy versus random movement) • whole brain: BOLD activity • FSL (FEAT)	• *OXTR* DNAm positively associated with activity in left STG, supramarginal gyrus, and right dACC. • Controlling for sex: *OXTR* DNAm positively associated with activity in the left STG and right dACC. No difference between men and women.

Vukojevic et al., 2014 Switzerland	• Healthy participants • Cases, n = 72(25 M) • Cases, median age 23 years	• Candidate: *NR3C1* - CpG3 • Saliva • Cell type composition: NR • Consideration of genotype: NR	• 3 T • fMRI task: N-back task–working memory task • Brain activation • SPM8	• Women: no significant associations between *NR3C1* CpG3 DNAm and brain activation during fMRI task. • Men: positive associations between DNAm at *NR3C1* CpG3 and brain activity related to successful recognition of previously seen pictures: right hemisphere pars orbitalis, right hemisphere pars triangularis of the inferior frontal gyrus; near superior temporal cortex; left hemisphere cuneus, left hemisphere pericalcarine; near superior frontal cortex.

Nikolova et al., 2014 United States	• Healthy participants, Discovery set n = 80(38 M); replication set: 96 (48 M) • Discovery set mean age 19.7 years; replication set 13.6 years	• Candidate: *SLC6A4-* 20 CpG sites in promoter region • Discovery: saliva • Replication: blood • Cell type composition: NR • Consideration of genotype: yes	• 3 T • fMRI task: emotional faces processing • BOLD - amygdala reactivity to threat (angry/fearful faces vs shapes). • SPM8	• Positive association between *SLC6A4* DNAm and left amygdala reactivity in response to threat stimuli; right amygdala did not reach statistical significance. • The CpG with the strongest association across both hemispheres is CpG14. • Findings consistent in discovery and replication sets

Dannlowski et al., 2014 Germany	• Healthy participants • Cases, n = 189(90 M) • Two independent samples: sample 1 n = 94(42 M); sample 2 n = 95(46 M) • Sample 1 mean age 36.9 years; sample 2 mean age 34.2 years	• Candidate: *SLC6A4* AluJb element – mean methylation of 8 investigated sites • Blood • Cell type composition: NR • Genotype: yes	• Field strength: NR • Structural, T1 • VBM-ROI volumes: hippocampus, anterior cingulate cortex, amygdala • VBM – whole brain. • VBM8/SPM8	• Positive association between AluJb DNAm in the *SLC6A4* gene and bilateral hippocampal grey matter volume in ROI analysis in both samples. In sample 1 peak coordinates cluster extended into the bilateral amygdala. This was not the case in sample 2, but was the case in the combined sample. • Whole brain analysis: positive association clusters where a positive association is also found: bilateral insula, right putamen, amygdala and caudate, and left superior occipital gyrus, bilateral hippocampus. • No areas showed a negative association between DNAm rates and grey matter volume.

Puglia et al., 2015 United States	• Healthy participants • Cases, n = 98(42 M) • Cases, mean age 22.8 years	• Candidate: *OXTR-* CpG site at position -934 relative to TSS • Peripheral blood mononuclear cells • Cell type composition: NR • Consideration of genotype: NR	• 3 T • fMRI task: emotional face matching task. • BOLD - ROI analysis • PPI/FEAT (FSL)	• Positive association between *OXTR* DNAm and BOLD in the left amygdala in Faces versus Ovals. No association in right amygdala. • Higher *OXTR* DNAm predicted decreased levels of functional connectivity on tasks involving affect appraisal and emotion regulation between right amygdala and brain regions involved in emotional regulation including insular cortex, cingulate cortex, orbitofrontal cortex as well as regions associated with face perception e.g fusiform gyrus. Left amygdala showed no significant voxels.

Ziegler et al., 2015 Germany	• Social anxiety disorder • Cases, n = 25(F) • Cases, mean age 28.8 years	• Candidate: *OXTR-* exon 3 • Blood • Cell type composition: NR • Consideration of genotype: yes	• 3 T • fMRI task: social phobia related verbal stimuli • Amygdala responsiveness • SPM8	• In SAD patients (n = 25 F): negative association between *OXTR* DNAm and amygdala responsiveness in social phobia vs negative words or neutral words.

Muehlhan et al., 2015 Germany	• Healthy participants • Cases, n = 74(45 M) • Cases (male), mean age 23.6 years; cases (female), mean age 23.2 years	• Candidate: *SLC6A4*- mean methylation score at the promoter • Blood • Cell type composition: NR • Consideration of genotype: yes	• 3 T • fMRI: resting state • SPM12	Positive associations between *SLC6A4* DNAm and amygdala RS functional coupling with nodes of the salience network:
				• Right amygdala: right SMA and left dACC, left putamen and left insula, right IFG, right putamen, right insula • Left amygdala: right dACC, left ACC, left SMA

Ursini et al., 2016 Italy	• Healthy participants • Cases, n = 141 • Age: NR	• Candidate: *BDNF* - rs6265 methylation status • Blood • Cell type composition: NR • Consideration of genotype: yes	• 3 T • fMRI task: N-back task, working memory • SPM8	Analysis carried out in 141 healthy subjects:
				• Positive association between *BDNF* rs6265 DNAm and left prefrontal activity • Val/Val genotype: greater DNAm is associated with reduced activity in the PFC (BA46) subjects with hypoxia related obstetric complications (assessed using the McNeil-Sjöström Scale) compared to those without. • Val/Met genotype: lower DNAm is associated with reduced activity in the prefrontal cortex (BA46) in subjects with hypoxia related obstetric complications compared to those without.

Haas et al., 2016 United States	• Healthy participants • Cases, n = 121(52 M) • Cases, mean age 21.3 years	• Candidate: *OXT* - average *OXT* DNA methylation across nine CpG cites • Saliva • Cell type composition: NR • Consideration of genotype: NR	• 3 T • Structural, T1 • fMRI task: Emotional perspective taking task; Emotion attribution task • VBM - SPM8	• Emotional perspective task: Negative association between *OXT* DNAm and activity in the R superior temporal sulcus during emotional perspective taking; • Emotion attribution task: negative association in the emotion attribution between *OXT* DNAm and activity in: right STS, right fusiform gyrus/middle occipital gyrus, right inferior frontal gyrus; left fusiform; • Negative association between *OXT* DNAm and grey matter volume in right fusiform gyrus

Resmini et al., 2016 Spain	• Cushing’s syndrome • Cases, n = 32; controls, n = 32 • Cases: mean age 45 years; controls, mean age 44 years	• Candidate: *FKBP5* - intronic regions • Blood • Cell type composition: NR • Consideration of genotype: yes	• 3 T • Structural, T1 • Hippocampal volume • FreeSurfer	• Cases: positive association between Intron 2 region 2 CpG3 DNAm and bilateral hippocampal volume

Ismaylova et al., 2017 Canada	• Healthy participants • Cases 40(14 M) • At time of blood sampling: 27 • At time of saliva/buccal sampling: mean 33.7 years	• Candidate: S*LC6A4* – promoter mean methylation score of 10 CpG sites • (blood/saliva/buccal) • Cell type composition: NR • Consideration of genotype: NR	• 3 T • fMRI task: Emotional face processing task • fMRI resting state • Structural, T1 • WB analysis and 10 ROIs grey matter volumes, functional connectivity • VBM/SPM12/Conn functional connectivity toolbox	• Positive associations between *SLC6A4* promoter DNAm and grey matter volumes, which varied by tissue type:
				- Blood: right superior frontal grey matter; - Saliva: left superior frontal gyrus; - Buccal cells: left superior frontal gyrus, left inferior frontal gyrus, right ACC.
				• Positive association between blood *SCL6A4* DNAm and RS connectivity between: RLP and bilateral frontal poles and SFG; RLP and left lateral occipital cortex • Positive associations between buccal *SLC6A4* promoter DNAm and RS connectivity between the RLP with right lateral occipital cortex and right angular gyrus, ACC, right frontal pole and mPFC • No significant associations were found for task-based fMRI

Wiemerslage et al., 2017 Sweden	• Orexigenia among healthy participants • Cases, n = 23(all M) • Cases, mean age 26 years	• Candidate: *KLF13* - cg07814318 probe methylation • Blood • Cell type composition: yes • Consideration of genotype: NR	• 3 T • fMRI resting state and task: virtual high or low-calorie food • WB • SPM8	• Negative associations between *KLF13* DNAm and neural activity in response to images high calorie food in comparison to images of low-calorie foods in: left claustrum and insula, bilateral cingulate gyrus, right precentral gyrus and claustrum, right MFG, SFG and medial FG. • Positive associations between DNAm and RS activity in the right caudate and putamen, left lingual gyrus and fusiform gyrus.

Ismaylova et al., 2018 Canada	• Fronto-limbic brain responses to sadness/fear • Cases, n = 96 monozygotic twins (21 M pairs) • Cases, mean age 15 years	• Candidate: *SLC6A4–* promoter CpG 5-14 • Saliva • Cell type composition: NR • Consideration of genotype: NR	• 3 T • fMRI task: Emotional face processing task • ROI functional connectivity • SPM8/CONN toolbox	• Sad condition: Positive association between *SLC6A4* DNAm and OFC activation. Positive association between methylation and functional connectivity between left amygdala and the ACC and the left amygdala and the right OFC • Fearful condition: positive association between *SLC6A4*methylation and functional connectivity between ACC and both the left amygdala and left insula.

Freytag et al., 2017 Germany/ Switzerland	• Healthy cohort n = 533(222 M) of whom 514 have imaging. • Mean age at MRI: mean 22.9 years; mean age at blood sampling 23.9 years	• Epigenome-wide: Illumina 450 array • Blood • Cell type composition: Methylomic profiling sample: yes; Replication cohort: none reported • Consideration of genotype: yes	• 1.5 T/3 T • Structural, T1 • Cortical thickness • FreeSurfer	• Independent component analysis (ICA) applied to decompose methylation profiles. • Negative association between the second independent component (ICA2) DNAm and cortical thickness. ICA2 DNAm partially mediates the relationship between chronological age and global cortical thickness. • Negative association between ICA2 and F6 (identified through exploratory factor analysis (EFA) of variation in cortical thicknesses); F6 accounts for 4% of variance in cortical thickness measures - spatial pattern comprising mainly temporal areas (loadings >0.3) with highest loadings in L and R temporal poles and entorhinal cortices.

The three studies that investigated associations with *OXTR* methylation measured DNAm at two different positions: one in exon 3 and the other two at a CpG site -934 base pairs upstream of the TSS which has consistent methylation across blood and brain tissues (Jack et al., 2012; Puglia et al., 2015; Rubin et al., 2016; Ziegler et al., 2015). While these studies all used fMRI, they also used different tasks: social phobia related verbal stimuli, the emotional face matching task, and the social perception task (where objects move in ways to suggest animacy or random movement). One study investigated *OXTR* DNAm in exon 3, which had previously been implicated in social cognition, in a patient population with social anxiety disorder (Ziegler et al., 2015). The other two investigated healthy participants; one of these was in the context of social perception and individual variability in social perception (displays of animacy) (Jack et al., 2012; Puglia et al., 2015).

Five papers analysed associations between *SLC6A4* methylation and neuroimaging features. They included fMRI (n = 4) and sMRI (n = 2). fMRI studies used resting state and/or emotional face processing tasks. Ismaylova et al. (2017) estimated DNAm from three different surrogate tissues: blood, saliva, and buccal cells and reported that right lateral parietal area (RLP) resting state connectivity with the lateral occipital cortex and frontal poles has a positive association with *SLC6A4* DNAm (Ismaylova et al., 2017). Muehlhan et al. (2015) found positive associations between functional coupling between amygdala and nodes of the salience network with DNAm of the *SLC6A4* promoter in blood (Muehlhan et al., 2015). In the emotional face processing task, two studies report positive association between *SLC6A4* methylation and amygdala reactivity/connectivity in response to threat/fear related faces. Nikolova et al. reported a positive relationship between saliva DNAm at *SLC6A4* and left amygdala reactivity to threat stimuli, while Ismaylova et al. reported that twins with higher saliva DNAm at *SLC6A4* display greater connectivity between ACC and left amygdala (Ismaylova et al., 2018; Nikolova et al., 2014). The two studies that used sMRI both found positive associations between DNAm at *SLC6A4* and grey matter volumes (Dannlowski et al., 2014; Ismaylova et al., 2017). However, they did not replicate each other by identifying the same brain regions as being associated even when using the same tissue to estimate DNAm.

## 4 Discussion

This systematic review of 60 studies involving approximately 6775 participants suggests that differential DNAm may be associated with MRI features of brain structure and / or function for conditions / diseases within the following categories: neurodevelopment and neurodevelopmental disorders; MDD and suicidality; alcohol use disorder; schizophrenia and psychosis; ageing, stroke, ataxia and neurodegeneration; post-traumatic stress disorder; healthy adults and socio-emotional processing. We found that 10 image features were associated with differential DNAm in two or more of the 60 studies. However, quantitative synthesis of DNAm-MRI associations was not possible due to the lack of consistency in DNAm findings, and / or heterogeneity in image features across studies. While the results presented here could provide new insights into the role of DNAm in health and disease across the life course, caution is required because the clinical and methodological heterogeneity of included studies was large. We identified the following sources of methodological heterogeneity and potential sources of bias: variable inclusion and characterisation of comparator groups in study designs; use of different tissues as a surrogate for brain; variation in methods for estimating DNAm and ascertainment of cell type composition; lack of control for genotype; and variations in image processing methodology and selected MRI features.

### 4.1 Study populations and comparator group validity

Twenty-six studies (43 %) did not include a comparator group, and some others recruited cases and controls but combined the groups in DNAm-MRI analysis or restricted DNAm-MRI analysis to one group only. These issues limit inference about disease association. Several aspects of study design that are fundamental to good epidemiology should be adopted in future DNAm-MRI studies to reduce potential sources of bias and enable maximum inference. These include detailed descriptions of participant selection, the population from which they were selected, and the method used to select. Not only is this important for understanding case definition, but also because research participation is influenced by social class, education, and ethnicity, and some of these factors may influence DNAm (Stafford et al., 2013). The same descriptive standards should be applied to comparator groups to ensure clinical validity, and attention should be given to matching comparator groups for ethnicity and age because both affect DNAm (Fraser et al., 2012; Heyn et al., 2013; Horvath, 2013; Talens et al., 2010).

### 4.2 Surrogate tissues for brain DNAm and cell composition

Surrogate tissues include blood and saliva buccal cell samples. This is important when interpreting data because of tissue and cell heterogeneity in DNAm patterns. Buccal cells, a major cell type found in saliva, have been proposed to have more validity than blood as a surrogate tissue for brain since they have a common embryological cell lineage, both being derived from the ectoderm germ layer (Smith et al., 2015). One study that ascertained DNAm from three sample types (blood, saliva, buccal - buccal cells and saliva samples are collected differently) found that DNAm obtained from non-blood surrogate tissues most strongly associated with brain processes in living humans in the context of fMRI study (Ismaylova et al., 2017). This is consistent with a previous study in postmortem brain tissue in animal models, which found that saliva and brain are more similar in their DNA methylation patterns than are blood and brain (Lowe et al., 2013). Surrogate tissues have heterogeneous cell compositions which can impact the DNAm signature of the sample: blood comprises lymphocytes, neutrophils, basophils, monocytes and eosinophils; and saliva contains predominantly buccal cells but can include leukocytes. Crucially, the compositions of both surrogates are influenced by disease status. Hence, adjustment for cell composition should be considered in DNAm analysis.

### 4.3 Candidate gene versus epigenome-wide approaches

Forty-three included studies in this review used a candidate gene analysis approach, with candidacy most often rooted in knowledge or hypotheses about the biological underpinnings of the target disease. The majority of candidate gene studies focused on promoter regions, which risks neglect of other important regulatory regions, because it limits research to currently understood genomic elements that have a clear role in gene expression regulation.

Although there are inherent sources of bias in the candidate approach, it has been suggested that candidate gene studies can provide a useful starting point for hypothesis construction for example by investigating candidates that have emerged from the GWAS literature (Nikolova and Hariri, 2015). However, we did not find that candidate selection was based on the GWAS literature in the majority of studies. For example, *SLC6A4* was the candidate gene in eleven studies. *SLC6A4* encodes a serotonin transporter responsible for serotonin re-uptake from the synapse, and is one of the targets for the serotonin reuptake inhibitor class of antidepressants. Despite the importance of this gene product in the pharmacology of depression, recent GWAS studies report no association between candidate polymorphisms at *SLC6A4* (or a number of other candidate genes) and major depression (Border et al., 2019). This suggests that candidate genes that encode important pharmacological targets are not necessarily aetiologically significant. This is not to say that the methylation status of these genes is irrelevant: for example, it could be useful for investigating medication response, or as a biomarker. Frodl et al. carried out an DNAm-MRI analysis with medication status as a covariate, and but found that this did not modify their findings regarding *SLC6A4* methylation and brain function in MDD patients and controls, while Booij et al. have reported differential methylation at the *SLC6A4* promoter in association SSRI exposure (Booij et al., 2015; Frodl et al., 2015). In a human population of MDD no difference was found in methylation between medication free subjects and those on medication in a post mortem study (Sabunciyan et al., 2012). These uncertainties suggest that further research into association between medication exposure and DNAm is warranted, and that hypothesis formation around candidate genes should be explicit in terms of theorised mechanism of disease or biomarker development. Further, mechanistic studies are crucial to aid our understanding of the interaction between DNAm and disease or therapy.

The rationales given for the selection of candidate genes for DNAm-MRI analysis were varied. Several studies selected candidates based on prior unbiased analyses in relation to phenotype (Kim et al., 2017; Ruggeri et al., 2015; Sadeh et al., 2016). For example, both *PP1MG* and *SKA2* were identified in EWAS analyses (Ruggeri et al., 2015; Sadeh et al., 2016), and *CACNA1C* was identified from a hypothesis free Methyl-Seq (Kim et al., 2017). Other candidate genes were chosen because they have a strong genetic link to the patient population being studied, such as *C9orf72* expansion in ALS-clinic patients and *FMR1* in FXTAS patients (McMillan et al., 2015; Shelton et al., 2017, 2016). For the most part candidate genes were selected because their gene products are considered key components of a pathway of relevance to a condition or phenotype studied. *NR3C1* and *FKBP5*, the genes that encode the glucocorticoid receptor and FK506 binding protein (which regulates glucocorticoid receptor sensitivity), are both involved in cortisol signalling and were selected in studies investigating stress response or stress related neuropsychiatric conditions such as PTSD or MDD, or in Cushing’s Syndrome where cortisol is known to be dysregulated. *BDNF*, which encodes for brain derived neurotrophic factor, was studied in association to MDD, PTSD and working memory. *COMT* encodes catechol-O-methyltransferase, an enzyme that degrades catecholamines such as dopamine, epinephrine and norepinephrine, was the candidate gene in studies investigating DNAm-MRI in the context of working memory in schizophrenia and in healthy participants. *OXTR* encodes the receptor for oxytocin and was selected in studies that investigated an aspect of emotional or social processing. *SLC6A4* encodes the serotonin reuptake transporter and is a target for the serotonin reuptake inhibitor class of anti-depressants. It was the selected candidate gene in studies of MDD and ADHD patients and emotional processing, and resting state amygdala activity.

Seventeen studies used an EWAS approach. There was no overlap between differentially methylated regions identified using this method versus the selected candidate gene approach for any of the phenotypic categories, which highlights the value of epigenome-wide approaches for unbiased investigation of the putative role of DNAm in brain structure and / or function. However, one study which employed an epigenome-wide approach to DNAm analysis identified *COMT* and *SLC6A3* as being negatively associated with right amygdala volume in neonates homozygous for *BDNF* rs6265 Met/Met allele (Chen et al., 2015). Both of these genes were selected as candidates in other studies but associations were reported in different populations with regards to age and clinical background, and with different image features (Muench et al., 2018; Ursini et al., 2011; Walton et al., 2014; Wiers et al., 2015). Many of the studies that utilised epigenome wide methods for measuring DNAm did not go on to report on individual loci. In future studies whole genome-based approaches could play an important role in generating novel candidates and pathways for future research that could provide mechanistic insight into pathogenesis.

### 4.4 Interindividual variability, effect of genotype, and temporal stability

Sources of variability that are crucial for understanding of how changes in DNAm associate with endophenotypes include interindividual variability which may be stochastic, genetic or environmental in origin, and temporal stability (Lancaster et al., 2018). Twin studies are useful for separating genetic and environmental contributions to variability, and several are included in the DNAm-MRI literature to date (Casey et al., 2017; Chen et al., 2018; Ismaylova et al., 2018). Twenty-four studies controlled for genotypic effects, for example by covarying for single nucleotide polymorphisms (SNPs), or for the first PC of the variance in genome wide genetic data. This is because variation in DNAm may be explained partly by common genetic variation, such as a SNP that removes a methylation site. Therefore, genotyping in DNAm-MRI analyses is necessary for determining whether epigenetic associations with brain structure and function are genotype-specific, or whether associations are independent of genotype.

The stability of some epialleles varies over time with dynamic methylation and demethylation at CpG sites occurring in typical development and ageing, and in response to environmental exposures (Lancaster et al., 2018). Only fifteen studies reported the timing of DNAm sampling in respect to MRI acquisition; we suggest that this information is included in future studies because it is required to compare study populations and interpret phenotypic associations. Longitudinal investigation could be used to establish the temporal stability of methylation at a genomic region or site, the impact of medication, and for assessing potential causal relationships.

All of the included studies carried out cross-sectional analysis and two additionally carried out longitudinal analysis. Three other studies performed mediation analysis to assess causal relationships (Casey et al., 2017; Freytag et al., 2017; Harms et al., 2017). Studies designed to investigate association or causation should report the magnitude of differential DNAm between groups since the described changes in DNAm are often small and the biological relevance of these remains uncertain. Ultimately however, differential DNAm-MRI data will need to be interpreted in the wider context of mechanistic studies of gene expression in order to make inferences about causality. For example, experiments that demonstrate changes in gene expression associated with the differential methylation patterns reported from DNAm-MRI studies would be an important first step in dissecting causality, although it is important to note that changes in DNAm may actually occur as a consequence of changes in gene expression.

### 4.5 Neuroimaging considerations

Structural and functional quantitative neuroimaging features have been included in this review, assessed using sMRI, fMRI and dMRI. Others have emphasised the importance of selecting image features that represent a deficient or pathological phenotype when investigating associations between DNAm and MRI (Lancaster et al., 2018). Five DNAm-MRI associations recurred in two or more studies (*SLC6A4* and amygdala reactivity; *OXTR* and amygdala reactivity; *BDNF* and PFC function; *FKBP5* and hippocampal volume; *COMT* and *PFC* function), and a narrative synthesis of these demonstrates that there were mixed directions of associations, which may be explained by the variation in where in a genomic region DNAm was measured and in which tissue, in tasks employed during fMRI and in participant characteristics. A variety of fMRI paradigms have been used to probe brain function in this field, such as resting state and emotional processing, and dMRI studies have used different diffusion gradient encoding schemes and strengths (b-values) to measure the mobility of water molecules in vivo. A further layer of complexity is added in terms of differences in image processing pipelines such as CIVET (http://www.bic.mni.mcgill.ca/ServicesSoftware/CIVET), FreeSurfer (https://surfer.nmr.mgh.harvard.edu) and SPM (https://www.fil.ion.ucl.ac.uk/spm) for sMRI; and voxel based methods e.g. TBSS (https://fsl.fmrib.ox.ac.uk/fsl/fslwiki/TBSS) or tractography methods e.g. probabilistic neighbourhood tractography (http://www.tractor-mri.org.uk/tractography) for analysis of dMRI (Ad-Dab’bagh et al., 2006; Anblagan et al., 2015; Bastin et al., 2010; Clayden et al., 2007, 2006; Fischl, 2012; Smith et al., 2006). In the future, it will be necessary to form consensus and harmonisation about the optimal acquisition protocols and analysis pipelines to allow findings from different groups to be combined more readily. Initiatives such as the ENIGMA consortium which aims to understand brain structure, function and disease using brain imaging and genetic data from groups around the world are an important step in this direction.

### 4.6 Strengths and limitations

This systematic review has several strengths. It was based on a predefined protocol and followed standard guidelines with rigorous screening of >3,400 articles and without language or publication year restrictions. Our analysis was based on 60 studies examining healthy individuals and a wide range of neurological and psychiatric diseases in more than 6,000 patients across the life course. This provided a detailed overview of a growing body of literature linking DNAm and MRI data types, and enabled us to identify the key sources of methodological variation that should be addressed as standards and conventions in DNAm-MRI analyses become established.

Our study also has limitations. First, there was substantial clinical and methodological heterogeneity which prohibited quantitative synthesis for any single phenotype category, or MRI feature. Second, in the absence of validated tools for assessing quality and risk of bias in DNAm-MRI analyses, we were unable to evaluate these issues quantitively.

### 4.7 Conclusion

There is some evidence of association between differential DNAm and human brain structure and / or function across the life course. However, varied methodologies used to acquire and analyse DNAm-MRI data prevent quantitative synthesis. The development of standards and conventions for studies linking DNAm with MRI data is required, with particular focus on: detailed case and comparator group definition; surrogate tissue type; adjustment for cell composition and common genetic variation; consistent approaches to DNAm estimation using whole genome approaches; and use of image acquisition protocols, analysis pipelines and image feature selection that best support pooled analyses that are likely to be required to achieve statistical power for EWAS-neuroimaging studies. In summary, this review has found that: differential DNAm is associated with image features of brain structure and function in health and disease across the life course using data from over 6,000 individuals; evidence that differential DNAm is associated with specific image features is modest; and it has identified specific sources of sample and methodological heterogeneity in existing DNAm-MRI analyses. We anticipate that identification of these will expedite rational development of analytic methods in this emerging field so that researchers might more rapidly design studies that support causal inference.
