# Supplementary material for: DNA methylation and brain structure and function across the life course: A systematic review
Source: Neurosci Biobehav Rev. 2020 Jun;113:133–56. doi: 10.1016/j.neubiorev.2020.03.007 (PMC7237884; doi:10.1016/j.neubiorev.2020.03.007)
Supplement: Supplementary file 2 [file mmc2.docx]

**Supplementary material**

**DNA methylation and brain structure and function across the life course: a systematic review**

Systematic review search strategy

**OVID (Embase and Medline)**

Epigenetics keywords

1. (DNA methyl* OR methylation Epigen* OR Methylome OR CpG OR CGI OR “bisulfite

adj1 (conversion OR treatment OR pyrosequencing OR sequencing)”).mp

OR

Headings:

2. DNA methylation/ OR Epigenomics/ OR CpG Islands/ OR Exp epigenesis, genetic/

3. 1 OR 2

Brain:

Keywords:

4. “small worldness” OR Connectome OR Connectivity OR Myelin* OR “white matter”

OR “Brain volume” OR “cortical connectivity” OR “graph theor*” OR topology OR

cereb* OR (Connect* adj5 (brain or neur*)).mp

Headings:

5. White matter/ OR Neural Pathways/ OR Brain Mapping/ or exp Brain/

6. 4 OR 5

MRI Keywords

7. (MRI OR DTI OR fMRI OR dMRI OR “Magnetic Resonance Imaging” OR “Diffusion

Tensor Imaging” OR “Diffusion Magnetic Resonance Imaging” OR “functional

Magnetic Resonance Imaging” OR dtMRI OR “diffusion tensor Magnetic Resonance

Imaging”).mp

8. Exp Neuroimaging/ OR Exp Magnetic Resonance Imaging/

9. 7 OR 8

10. 3 AND 6 AND 9

**Web of Science**

TS = (("DNA methyl*" OR methylation or Epigen* OR Methylome OR "CpG Island$" OR

"CpG" OR “bisulfite adj (conversion OR treatment OR pyrosequencing OR sequencing)”)

AND ("small world$" OR "graph theor*" or topology or connectome OR connectivity OR

connect* adj (brain OR neur*) OR "brain volume" OR "Neural pathways" OR "Brain

Mapping" OR "Cerebral Cortex" OR "cortical connectivity" OR Brain OR "Visual Cortex" OR

“White matter” OR “Myelin$” OR hippocamp* OR amygdal* or hypothalam* OR thalam*

OR cereb* OR cortex OR cortical OR Neuro*) AND ("Magnetic Resonance Imaging" OR

"Diffusion Tensor Imaging" OR "Diffusion Magnetic Imaging" OR MRI OR DTI OR

Neuroimaging OR "functional MRI" OR fMRI OR "functional Magnetic resonance imaging"

OR “functional MRI” OR dtMRI OR dMRI OR “diffusion tensor Magnetic resonance

imaging”))

**Scopus**

TITLE-ABS-KEY ( ( "DNA methyl*" OR methylation OR epigen* OR methylome OR "CpG

Island$" OR "CpG" OR "bisulfite adj (conversion OR treatment OR pyrosequencing OR

sequencing)" ) AND ( "small world$" OR connectome OR topology OR "graph

theor*" OR connectivity OR "connect* adj (brain OR neur*)" OR "brain

volume" OR "Neural pathways" OR "Brain Mapping" OR "Cerebral Cortex" OR "cortical

connectivity" OR brain OR "Visual Cortex" OR "White

matter" OR "Myelin*" OR "hippocamp*" OR "amygdal*" OR "cortex" OR "cortical" OR

"Neuro*" OR hypothalam* OR thalam* OR cereb* ) AND ( "Magnetic Resonance

Imaging" OR "Diffusion Tensor Imaging" OR "Diffusion Magnetic

Imaging" OR mri OR dti OR neuroimaging OR fmri OR "functional Magnetic Resonance

Imaging" OR "functional MRI" ) )
